# Causes of irritant contact dermatitis after occupational skin exposure: a systematic review

**DOI:** 10.1007/s00420-021-01781-0

**Published:** 2021-10-19

**Authors:** Gitte Jacobsen, Kurt Rasmussen, Anne Bregnhøj, Marléne Isaksson, Thomas L. Diepgen, Ole Carstensen

**Affiliations:** 1 Department of Occupational Medicine, Danish Ramazzini Centre, Regional Hospital West Jutland, University Research Clinic, Herning, Denmark; 2 Department of Occupational Medicine, Hospital South West Jutland, University Hospital of Southern Denmark, Esbjerg, Denmark; 3 Department of Dermatology, Aarhus University Hospital, Aarhus, Denmark; 4 Department of Occupational and Environmental Dermatology, Skåne University Hospital, Lund University, Malmö, Sweden; 5 Department of Clinical Social Medicine, Occupational and Environmental Medicine, University Hospital Heidelberg, Heidelberg, Germany

**Keywords:** Occupational contact dermatitis, Irritant contact dermatitis, Hand eczema, Skin exposure, Prognosis

## Abstract

**Purpose:**

Irritant contact dermatitis (ICD) is a major cause of occupational disease. The aim was to review the relation between exposure to occupational irritants and ICD and the prognosis of ICD.

**Methods:**

Through a systematic search, 1516 titles were identified, and 48 studies were included in the systematic review.

**Results:**

We found that the evidence for an association between ICD and occupational irritants was strong for wet work, moderate for detergents and non-alcoholic disinfectants, and strong for a combination. The highest quality studies provided limited evidence for an association with use of occlusive gloves without other exposures and moderate evidence with simultaneous exposure to other wet work irritants. The evidence for an association between minor ICD and exposure to metalworking fluids was moderate. Regarding mechanical exposures, the literature was scarce and the evidence limited. We found that the prognosis for complete healing of ICD is poor, but improves after decrease of exposure through change of occupation or work tasks. There was no substantial evidence for an influence of gender, age, or household exposures. Inclusion of atopic dermatitis in the analysis did not alter the risk of ICD. Studies were at risk of bias, mainly due to selection and misclassification of exposure and outcome. This may have attenuated the results.

**Conclusion:**

This review reports strong evidence for an association between ICD and a combination of exposure to wet work and non-alcoholic disinfectants, moderate for metalworking fluids, limited for mechanical and glove exposure, and a strong evidence for a poor prognosis of ICD.

**Supplementary Information:**

The online version contains supplementary material available at 10.1007/s00420-021-01781-0.

## Introduction

Irritant contact dermatitis (ICD) is a common disease. General population studies have reported incidences of non-specific hand eczema (HE) of around 5.5/1000 person-years, point prevalence around 4%, 1 year prevalence around 10%, and a lifetime prevalence of 15%, with ICD being the most prevalent type and the highest frequency of self-reported HE being among young women (Thyssen et al. 2010). Contact dermatitis (CD), mainly HE, is the most frequently recognized industrial injury in Denmark with up to 2000 annual cases recognized and compensated by the Danish National Board of Industrial Injuries (AES 2016). Allergic cases constitute 30% of the recognized cases of occupational contact dermatitis (OCD), while around 70% are caused by irritant exposures, mainly wet work, but also to use of occlusive gloves, exposure to fresh food, detergents, various oils, and dirt. The majority, around 70%, of the recognized cases of occupational ICD (OICD) in Denmark are in females (Caroe et al. 2014).

The majority of studies with clinical estimations of the incidence of occupational skin diseases (OSD) are based on occupational disease registers in which the reported incidences of OCD may vary from 5 to 19 cases per 10,000 fulltime worker years (Diepgen 2003; Diepgen and Coenraads 1999; Keegel et al. 2009; Lushniak 2003). These national registers are usually incomplete due to underreporting of the diseases, and registers are often not fully comparable because of differences in reporting practices across countries. In Denmark, underreporting of OCD among hairdressers was estimated in a register-based questionnaire study that found that only 21% of hairdressers with HE were reported to the National Board of Industrial Injuries (Lysdal et al. 2012). In the United Kingdom, data on OCD cases showed that occupational physicians reported substantially more cases than dermatologists, with overall incidences of OCD per 10,000 worker being reported by 5.1 and 0.7 (from 1996 to 2001) (McDonald et al. 2006) and 2.6 and 0.7 (from 2002 to 2005) (Turner et al. 2007).

The Saarland study from Germany reported an overall annual incidence of OSD of 6.8 per 10,000 workers in 16 occupational groups, based on 263 notifications of confirmed OSD, where 75% involved ICD. The annual incidences of OSD ranged from 1.5 to 48 per 10,000 highest among hairdressers, followed by bakers/pastry cooks, cooks, and nurses (Dickel et al. 2002a). Similar results were reported in a study from Northern Bavaria of 3,097 patients with OSD from 24 occupational groups; ICD was seen in 57%, and in combination allergic contact dermatis (ACD) in 15%. The annual incidence of ICD was 4.5 per 10,000 workers (Dickel et al. 2002b).

A Danish survey based on 758 recognized notified cases also found the highest overall annual incidence rates of OICD at 51.7 and 46.6 per 10,000 workers among high-exposed bakers and hairdressers (Skoet et al. 2004).

Currently, there is a general lack of knowledge, and no systematic reviews regarding the exposures sufficient to cause irritant eczema or aggravate pre-existing eczema. Previously only few systematic reviews on OICD have been published, mainly focusing on treatment and prevention (Bauer et al. 2018; Nicholson et al. 2010). Until recently, only a few epidemiological studies of OICD have been published, and most of our knowledge about OICD has been derived from clinical case reports and clinical studies of groups of patients with little differences in exposure(Diepgen and Coenraads 1999).

Our aim was to perform a systematic review to present:

- An assessment of the risk of ICD in relation to the character, level, severity, and duration of the exposure (dose–response relation) to occupational irritants,
- A description of the time of onset of the disease in relation to exposure and possible threshold values (lower limit of effect).
- And an assessment of the prognosis of ICD as well as the impact of continuous exposure on prognosis and the effect of cessation of exposure.

## Background

### The outcome: definition of ICD and irritant HE

CD has been defined by the European Society of Contact Dermatitis (ESCD) as an eczematous local inflammatory skin reaction caused by direct and usually repeated exposures, to harmful objects or chemicals, which, depending on location of contact, can occur anywhere on the body. CD is clinically characterized by redness of the skin, itching papules, or vesicles, but may vary from slight hyperkeratosis to fissures, swelling, scaling, and oozing. Histopathological CD is characterized by inflammation in the superficial parts of the skin, i.e., the outermost layers of the dermis with involvement of the epidermis. Healing is without scars. Besides ICD, there are three other forms of CD, with the most important being ACD, which is characterized by an acquired hypersensitivity with involvement of “allergen-specific” T cells as mediators of the inflammatory skin reaction. Another type of CD is photocontact dermatitis, which is the result of an interaction between a harmful substance in the skin and ultraviolet radiation. This can be either photoallergic contact dermatitis, which is an immunological disease much like contact allergy but where UV is required, or a phototoxic dermatitis, a non-allergic reaction that can happen to anyone exposed to the chemical in question and UV radiation. Finally, CD may be a type 1 allergic reaction, contact urticaria based on IgE specific antibodies, and protein contact dermatitis (ESCD 2020; Rustemeyer et al. 2011).

ICD is the most common variant of CD, and ICD has traditionally been defined as a local inflammatory non-specific reaction of the skin without requiring prior sensitization of the immune system, following single or repeated exposures to an irritant, which can be defined as any agent, psychical or chemical, capable of producing cellular perturbation if applied for sufficient time and in sufficient concentration (Ale and Maibach 2014).

ICD is not a clinical entity, but rather a spectrum of diseases, with different clinical presentations and etiological factors. Clinical entities have been described by some authors to encompass a classification scheme of *10 main phenotypes* based on both morphology and mode of onset, with the following seven most relevant in an occupational setting:

(1) Acute ICD, (2) delayed acute ICD, (3) irritant reactions, (4) cumulative (chronic) ICD, (5) traumatic ICD, (6) acneiform ICD, and (7) friction ICD (Ale and Maibach 2014).

Irritant HE is the major location of ICD, and as for CD and ICD in general, no gold standard for HE diagnosis or classification of HE exists (Agner et al. 2015).

A classification of HE recommended in guidelines (Diepgen et al. 2015) from the ESCD is based on a combination of etiology and morphological signs, with the following subgroups of HE: ICD, ACD, atopic HE, contact urticaria (CU)/protein CD, vesicular/pompholyx endogenous HE, and hyperkeratotic and endogenous HE, with further combination of diagnoses, e.g., ICD with and without atopic HE and combined ICD and ACD. The subgroup of ICD requires a documented exposure of the hands to an irritant being quantitatively likely to cause CD, with no relevant contact allergy, while ACD and allergic CU require relevant exposures to contact allergens identified by patch or prick tests (Agner et al. 2015; Diepgen et al. 2009; Diepgen et al. 2015).

No specific diagnostic test exists for ICD, and a diagnosis is made clinically as an exclusion diagnosis based on no findings of allergic CD and a temporal relationship to a history of supposed relevant irritant exposures (Friis et al. 2014).

### The exposures

Occupational ICD has been described in relation to exposure to various chemicals, soluble oils/metalworking fluids (MWF), wet work, detergents, occlusion by gloves, foods, exposure to plants, and mechanical friction.

The most prevalent exposures are wet work in various industries, including healthcare, cleaning, hairdressing, or exposure to chemical substances, e.g., MWF.

The criteria and definition of wet work, are not well defined internationally. The German Approved Code practice TRGS 401 (technical rules of hazardous substances) in 1996 defined criteria for wet work as regular work > 2 h/day, with the hands exposed to a wet work environment or regular use of occlusive gloves for the same period or frequent or intensive handwashing (BAuA 2008). A definition of frequent or intensive handwashing is not given in the TRGS, but has been suggested in the literature to about 20 times/day (Diepgen and Coenraads 1999).

Non-occupational exposures to irritants in the home environment including hobby activities may be of relevance in both research and clinical cases. Examples are domestic activities like dishwashing, cleaning, and childcare, but also hobby activities like mechanical repair, construction work, and use of different types of glue, etc. (Ibler et al. 2012; Meding et al. 2013; Thyssen et al. 2010; Visser et al. 2014a).

### Atopy and other genetic factors

Atopic disposition, especially a personal history of childhood atopic dermatitis (AD), has been described as a well-known risk factor for increased susceptibility for irritant HE/ICD (Diepgen and Coenraads 1995). A history of AD has been reported to increase the odds ratio (OR) of developing HE by a factor 3 in both wet and dry work (Coenraads and Diepgen 1998; Nilsson et al. 1985).

Other relevant factors include variations/polymorphisms of genes involved in the skin barrier function and inflammatory mediators including cytokines and polymorphism of gene coding for the epidermal protein filaggrin, which, as a structural protein in the epidermis, is important for the formation of the epidermal skin barrier (Davis et al. 2010; de Jongh et al. 2008; Diepgen et al. 2015; Kezic et al. 2009; Landeck et al. 2012; Visser et al. 2013; Visser et al. 2014a).

### Previous hand eczema

Previous episodes and early onset of HE are also well-known risk factors for the development of HE, including ICD. This probably relates to individuals with increased susceptibility due to inborn characteristic of the skin, including atopic skin disease, but possibly also due to behavioral patterns regarding habits of skin protection and handwashing (Diepgen et al. 2015; Mortz et al. 2014).

### Gender, age, and smoking

ICD including OICD is almost consistently reported to be more frequent in women, especially young women. However, no gender difference regarding irritant reactivity has been confirmed in experimental studies, and as many female-dominated occupations, e.g., HCW, cleaners, and hairdressers involve more extensive wet work along with females spending more time with wet work at home, the difference between male and females is suspected to be due to differences in occupational and non-occupational exposure to irritants (Anveden Berglind et al. 2009; Meding 2000; Meding et al. 2016; Thyssen et al. 2010).

Skin susceptibility to irritation decreases with increasing age. Population studies on hand eczema have consistently shown a trend toward declining frequency with age, especially among women (Slodownik et al. 2008; Thyssen et al. 2010).

The relation between smoking and HE has recently been evaluated in a review including 20 epidemiological studies with conflicting and inconsistent results. Approximately half the studies showed an increased prevalence and/or severity of HE in smokers, while the other half reported no association, although a protective effect of smoking was only reported in one study (Sorensen et al. 2015).

## Methods

### Literature search

The systematic review is based on PRISMA (Preferred Reporting Items for Systematic Reviews and Meta-Analyses) (Moher et al. 2009), a revision of the QUOROM statement (quality of reporting metanalysis) (Moher et al. 1999). The literature search was initially performed in the following international databases: PubMed, Embase, Web of Science, and OSH-update (HSELINE, NIOSHTIC, CISDOC, and RILOSH) in November 2015. The search was later updated in PupMed until 3 March 2020. The literature search was broad and included combinations of Mesh or text search terms for outcome (contact dermatis, hand dermatoses, dermatitis, and occupational dermatitis), irritative skin exposure (irritants, phototoxic, wet work, detergent, cutting fluid, and industrial oils), and work relation (occupational exposure, occupational diseases, occupation, and industry). Details on the search strategy for the different databases are presented in table S1 of the Online Supplementary Material.

We included papers with abstracts published in English, Danish, and German from January 1980 to March 2020. The first author performed the initial title screening based on the title of the articles.

In further screening of abstracts and articles, each paper was reviewed independently by two members of the group. The two reviewers had to agree on the inclusion criteria of each paper before enrollment for dataextraction.

### Inclusion criteria

Preselection of articles on associations between irritant exposures and ICD was based on the following eligibility criteria:

1. Original epidemiologic peer-reviewed studies on occupational exposure to irritants and outcome of ICD.
2. Study design included case–control, cross-sectional, and follow-up studies. Case studies, case series (patient populations reporting on proportion of ICD and ACD), meta-analyses, and reviews were excluded.
3. The included studies had to provide qualitative or quantitative exposure contrast either within the exposed group or include a control group without exposure.
4. Regarding outcome of ICD, we included studies which reported ICD or irritant changes resembling mild cases. We included studies with clinical assessment including patch test, but also studies with less diagnostic accuracy, i.e., studies with clinical examinations but no patch tests. Studies based on self-reported outcome of HE were also included if the studied association was presumed to be to irritant work exposures.
5. Studies with main focus on AD, ACD, and CU, without indication of ICD or exposure to irritants, were excluded.
6. Regarding studies on prognostic factors of ICD, we only included follow-up studies of cohorts with clinically verified occupational ICD. Studies with sole focus on treatment or prevention, e.g., use of barrier creams, were excluded.

### Data assessment: data extraction, quality, bias, and confounding

From each study, we extracted core information relevant for a description of relations between occupational exposures and diagnosis or symptoms of ICD and also on individual risk factors and prognosis of ICD, assessment of internal validity, and the overall quality of the studies (Table S2 in Online Supplementary). Two reviewers independently extracted the information, and disagreements were resolved by discussion.

We systematically assessed all studies to grade for eight quality dimensions resembling risk of bias and confounding (Beer et al. 2015). Quality factors were related to: study design (3 parameters), exposure assessment (2 parameters), outcome (2 parameters), and confounding (1 parameter).

Quality factors were dichotomized in each study by a score of 0 (low quality/high risk) or 1 (high quality/low risk) according to the following criteria:

i. Study design: cohort study or case–control study with population or hospital control vs case–control studies with convenience controls and cross-sectional studies
ii. Size of study, number of participants: > 75 cases vs < 75 cases
iii. Response rate > 60% vs < 60% (in cohort studies defined as proportion of baseline participating at follow-up)
iv. Source of exposure information: non-self-reports vs self- reports
v. Exposure measure: quantitative or semi-quantitative vs qualitative
vi. Source of diagnosis: hospital vs surveillance schemes, questionnaire, or not well-defined sources
vii. Diagnosis: well-defined diagnostic criteria for ICD vs other criteria
viii. Possible confounding: age, gender, atopy in adjusted analyses or by matching being taken into account vs age, gender, and atopy not being taken into account.

Based on the above *quality factors* and a discussion on the overall quality of the paper, each study was assigned a grade from 1 to 5, corresponding to highest, high, medium, low, and un-acceptable quality (Table S3 of the Online Supplementary).

When the study did not provide any kind of risk estimate, we calculated prevalence ratios, relative risk, or the OR based on the available data—whenever feasible calculations has been marked with asterisks (*) in the text and tables.

We rated the overall level of evidence of a *causal association* between a given exposure and a specific outcome according to a classification system established by The Scientific Committee of the Danish Society of Occupational and Environmental Medicine, and adopted by the Danish Working Environment Research Fund (details in S4 of the Online Supplementary Material).

The following categories were used:

- +++strong evidence of a causal association
- ++moderate evidence of a causal association
- +limited evidence of a causal association
- 0 insufficient evidence of a causal association or evidence suggesting lack of a causal association

–evidence suggesting lack of causal association.

For studies publishing multiple articles on the same issue, we included each paper if it provided additional information of the relation between exposure and OICD.

## Results

### Selection of papers

**Fig. 1:**
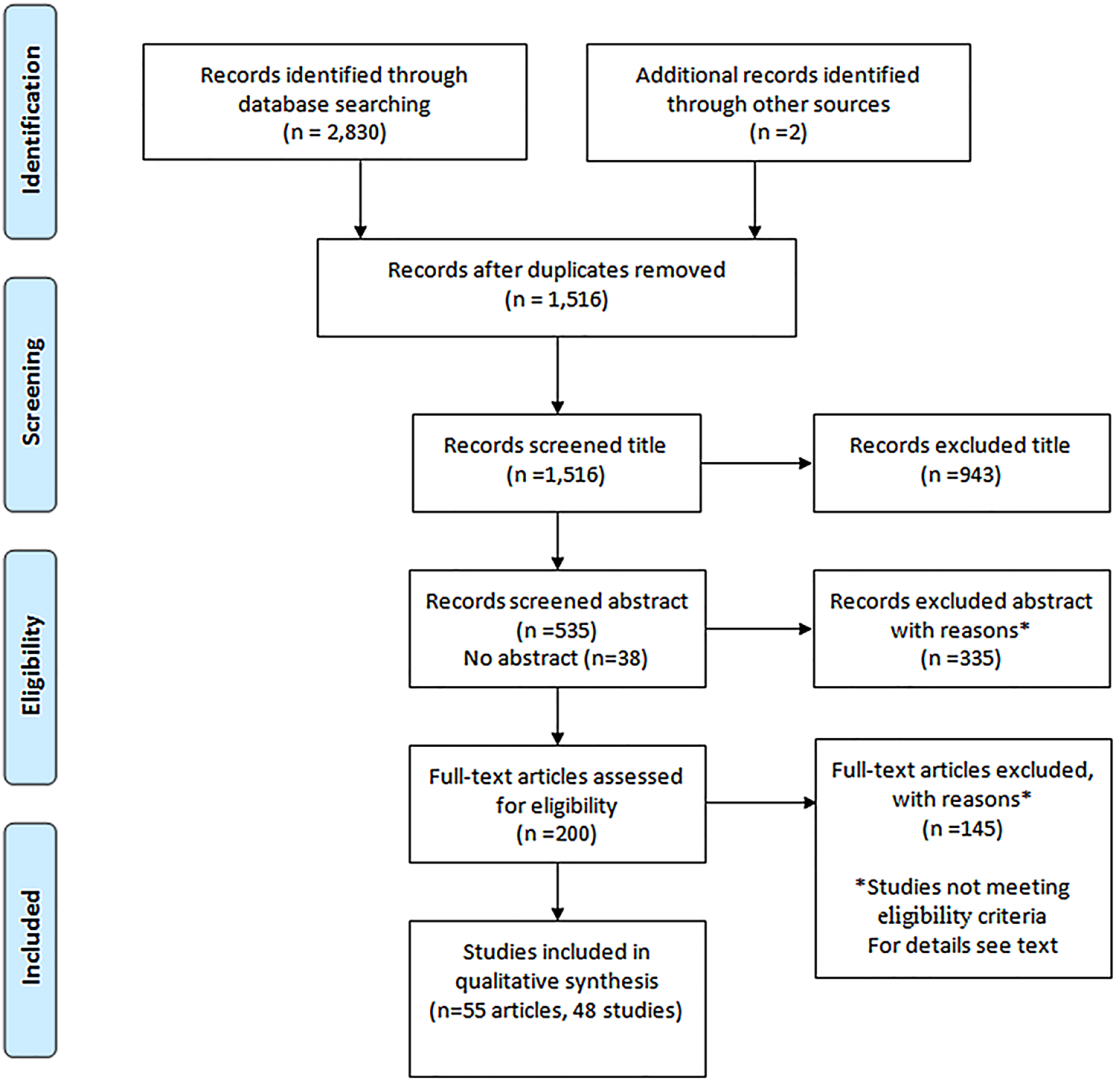
Flowchart of the inclusion of studies

The literature search, after removal of duplicates, resulted in a total of 1514 articles. In all, 1000 papers originated from PubMed, 218 from Embase, 189 from OSH-update, and 107 from Web of Science. Based on the title, 943 articles were excluded, and 38 articles without an abstract were excluded. The remaining 533 articles were evaluated based on the abstract, which resulted in exclusion of 335 articles. 198 articles were evaluated by reading of full text and 145 articles did not meet inclusion criteria. In addition, two snowball articles were included based on references in included articles and reviews, resulting in 55 epidemiological papers from 48 studies (Fig. 1). The large number of articles excluded by title and abstract were due to the broad literature search, which resulted in numerous articles with titles related to non-relevant outcome and exposures, e.g., ACD due to specific allergies and abstracts for studies clearly not meeting inclusion e.g., case series of patient populations.

Diagnostic outcome based on a clinical diagnosis with patch testing that made it possible to distinguish ICD from ACD was measured in 11 studies (Table 1). Eleven studies relied on clinical diagnosis without patch test (Table 2) and 16 studies relied on self-reported outcome (Table 3). Altogether 11 studies involved prognosis of ICD (Table 4).

**Table 1:** Occupational exposure, outcome based on clinical examinations, and patch tests

Author Year Country	Study design and population	Exposure and exposure assessment	Outcome diagnostic criteria	Covariates accounted for	Results PR/RR/OR
Wet work occupations					
Healthcare workers					
Held et al. (2001) Denmark	Prospective 10- week non-RCT intervention study of nurse student from 2 schools Intervention/controls: Baseline: *N* = 61/46 F-up: 54/40	Self-rep. daily exp: wet work, gloves, moisturizers, disinfectants Intervention: educational program	HE/skin irritation, graded mild (1–5 points), moderate/severe (> 5 points) Measurements of TEWL during f-up	Age, gender, AD, previous HE, domestic exp. nickel allergy	CIP HE/skin irritation: intervention vs controls 39%/48% Adj. OR: AD and baseline skin problems: **4.89** (1.16–20.64) Aggravation of skin problems, use/non-use of hand disinfectants: **6.13** (1.11–38.9)
Stingeni et al. (1995) Italy	CS study of hospital empl. Questionnaires, clinical exam. incl. patch and prick skin tests *N* = 1,301	Self-rep. exp: disinfectants, latex gloves & cleaning products Proxy of exp. contrast. department /job category	HE: objective signs, history and results of skin test ACD: relevant pos. patch test ICD: correlation of exp. to irritants and onset of HE	Age, gender, atopy	PP HE 28.1%; OCD 21.2%, ICD 20.1%, ACD 1.1% PR* OCD: Dep. internal medicine vs: radiological 8.37* (2.74–25.6); vs lab 2.22* (1.46–3.36); vs surgical 1.57* (1.26–1.96) PR* OICD: cleaners vs doctors 2.97* (1.97–4.46); cleaners vs nurses **1.20** (0.92–1.55); nurses vs doctors 2.39 (1.66–3.45)
Hairdressers					
Guo et al. (1994) Taiwan	CS study hairdressing shops. Interviews and clinical exam. incl. patch test *N* = 36 stylists, 62 apprentices	Self- rep: Duration hr/week total of work tasks apprentices/stylists, incl. shampooing: 15.1/0.01; waving: 12.6/6.9; drying: 7.6/21.9; cutting: 0/8.9 Glove wearing at work tasks	HE, exam. + photographs HE classified dry irritant/MP type and eczema of the fingers 3 scale severity of HE	Age, gender, atopy, sensitization allergens/nickel: 44%/28%	Apprentices vs stylists, HE: PP, PR*: All HE 98.3% vs 56.6%, PR* **1.69** (1.28–2.23); Moderate/severe HE: 69.3% vs 27.8%, PR* **2.50** (1.44–4.34) Dry irritant type:69.4% vs 36.1%, 1.9* (1.2–3.1) Eczema fingers: 22.6% vs 16.7%, all with positive patch tests
Food-related industry					
Teo et al. (2009) Singapore	CS study of kitchen and service worker at 26: restaurants *n* = 254; catering *n* = 30; fast food outlets* n* = 51 Questionnaire, clinical exam, patch/prick test	Self-rep. handwashing freq, exp. to detergents, chemicals, raw food Hand wash > 20 times/day: 36%	OICD: rash after start of job Patch test of subgroup with OCD and suspected ACD CU: prick test	Age, gender, atopy, ethnicity, compliance with glove use	12 months PP OICD 10% Adj. PR: Handwashing > 20 times/day: **2.8 (**1.4–5.7); Contact with squid: **2.6** (1.2–5.5) Atopy: **3.9** (1.9–8.0)
Tacke et al. (1995) Germany	Register study of recognized cases of OSD in 3 yrs Clinical exam. incl. patch test Bakers:107 (5,611 population) Confectioners: 31 (3,691) Cooks: 79 (23,252)	Occupation proxy of exposure Median self-rep exp. time months: Bakers 26, confectioner 26, cooks 43	OISD (OICD) definition: severe or relapsing dermatosis necessitating stop of all occupational activities related to specific exposures	Age, gender atopy	OICD % of cases: 70% in bakers, 87% in confectioners and 84% in cooks OICD RR* Baker vs cook: **4.73** (3.40–6.58); vs confectioner: **1.84** (1.19–2.85); PR* Confectioner vs cook: **2.58** (1.58–4.09)
Kavli and Moseng (1987) Norway	CS study at 2 fish factories Workers: fish-stick *n* = 122 vs fillet production* n* = 102 Questionnaire and clinical exam	Fish-stick workers: packing of frozen fish-blocks, flour-dust, cardboard boxes, gloves Filet workers: cutting of fish fillets without use of gloves	ICD ACD and CU: Patch test and prick test	Age, gender, atopy	PP ICD fish-stick workers/fillet workers 5.3% (*n* = 7)/ 2.4% (*n* = 3), PR* 1.95 (0.52–7.35) PP CU fish-stick workers/fillet workers: 7.4%/2.4% ACD: None occupational
Gloves					
Stingeni et al. (1996) Italy Study subpopulation of Stingeni et al. (1995)	CS study of hospital employ. Questionnaires, interviews, clinical exam. incl skin tests when glove reaction/atopy *N* = 922	Latex gloves Department and job proxy of exp. (nurse, doctor, cleaners, laboratory workers, radiology assistant)	ICD: HE, neg. patch and prick test for relevant allergens, positive use test latex gloves	Age, gender, atopy	PP HE 13.6%; ICD 13%: males 7.2%, females 17.9% ICD PR*: cleaners vs nurses **1.78** (1.15–2.74); cleaners vs doctors: **2.59** (1.48–4.52) HE PR* departments: internal vs lab: **1.82** (1.00–3.30), internal vs surgical: **1.83** (1.29–5.59)
Metalworking fluids and oils					
de Boer et al. (1989) The Netherlands	CS study metalworkers at 10 factories, exp. MWF. Interviews, clinical exam. Incl, patch test when present/past eczema *N* = 286	MWF (W-MWF* n* = 181, O-MWF* n* = 36, both *n* = 69) Factory visits: handling of MWF, extensive individual exp. Frequent exp: > once per hr (78%) Infrequent exp: < once per hr	Clinical Dermatitis: Minor (slight erythema, chapping), Major (eczema or widespread erythema, induration) ICD: Dermatitis + neg. patch tests	Atopy, allergy (2.8% ACD excluded analysis)	PP: CD 14%, ICD 11%, irritant skin changes 58% Irritant skin changes PR* Frequent vs infrequent W-MWF: **2.90** (1.22–6.92) Frequent W- MWF vs frequent O-MWF: **2.34** (1.29–4.25)
Jee et al. (1986) Taiwan	CS study of female workers of Ball Bearing factory vs zipper-manufacturing company Clinical exam. Incl. patch test *N* = 79/263	Kerosene (for degreasing) Semi-quantitative, expert judgement: heavy exp: 5 h a day/ light exp. < 5 h a day	Clinical Dermatoses i) Erythema ii) Eczema iii) Defatting dermatitis	Age, gender	Dermatoses/Eczema exp. vs controls 84%/15% vs < 1% (p < 0.001) Dermatoses/eczema, high vs low exp.: PR* 1.17 (0.97–1.41) / 1.56 (0.54–4.47)
Fischer and Rystedt (1985) Sweden	CS study of workers at a hard-metal factory. Interview, clinical exam. incl. patch tests *N* = 776	Present and previous work with grinding vs other work activities Exp. assessment from observed work task	Present or previous HE/Dermatitis or irritant reactions	Age, gender, atopy, contact sensitivity to relevant allergens	Current or previous skin reactions: grinders vs other groups, PR*: HE: 0.88* (0.64–1.20) Irritant reactions: 3.66* (2.73–4.90) Current or previous skin reactions: wet oil grinding vs dry grinders & others, PR*: HE: 1.93* (1.46–2.56) Irritant reactions: 3.38* (2.33–4.88)
Fibres					
Kiec-Swierczynska and Wojtczak (2000) Poland	CS study at 6 factories manufacturing ceramic fibers Ref: Metal press operators, dyers or seamstresses in same area *N* = 226 /43	127 individual dust measurements. Range total dust 0.2–33.9 mg/m^3^ Patch test % fibers > 3 μm Thermowool:: no fibers L-2 (polish): 6.3% L-3 (polish): 11.1%	ICD diagnosed clinical Acute ICD: transient Chronic ICD: Persistent erythema, telangiectasias on face neck, trunk Patch test of relevant allergens and irritative response to ceramic fibers	Age, gender	ICD: 48.2%, vs 7%, PR* 6.91* (2.30–20.8) Acute ICD: 30.5% vs 0%, RD* 0.31* (0.25–0.37) Chronic ICD: 26.1% vs 7%, PR* 3.74* (1.22–11.4) Patch test confirmed irritative activity of fibers in 19.5% Irritantancy correlated to of fibers > 3 μm All exp. reported strong itching of skin

Diagnoses: *ACD* Allergic contact dermatitis, *AD* Atopic dermatitis, *CD* Contact dermatitis, *CU* Contact urticaria, *HE* Hand eczema, *ICD* Irritant contact dermatitis, *OCD* Occupational contact dermatitis, *OICD* Irritant occupational contact dermatitis, *OSD* Occupational skin disease. Exposures and confounders: *CS*_*2*_ carbon disulphide, *H*_*2*_*SO*_*4*_ sulfuric acid, *exp* exposure, *HCW* Healthcare workers, *exp* exposure, *MWF* Metal working fluids; *W-MWF* water-based MWF, *O-MWF* oil-based MWF, *TDI* toluene diisocyante. Study characteristics: *adj* adjusted, ass association, *CC* case–control, *CIP* Cumulative incidence proportion, *CS* cross-sectional, *diff* differences, *DRR* dose–response relation, *exam* examination, *f-up* follow-up; , *freq* frequency, *hr* hour(s) , *incl* included, *neg* negative, *NR* not reported, *NS* non-significant, *OR* odds ratio, *PP* Prevalence proportion, *PR* Prevalence ratio, *S* significant, *RR* risk ratio, *IRR* incidence rate ratio, *RD* risk difference, self-rep self-reported, *TEWL* Transepidermal water loss, *vs* versus, *yr* year

*Calculated from data provided in article.

**Table 2:** Occupational exposure, outcome based on clinical examinations, but without patch tests

Author Year Country	Study design and population	Exposure and exposure assessment	Outcome diagnostic criteria	Covariates accounted for	Results PR/RR/OR
Wet work occupations					
Healthcare workers					
Callahan et al. (2013) USA	6-month f-up study of HCW, hand wash ≥ 8 times/day. Questionnaire and patch test with irritants Clinical exam.—1 month interval *N* = 102	Self-rep baseline daily freq. of handwashing, mean (SD): 12 (5.7) Season (cold and warm)	ICD hands clinical diagnosis ICD classified active dermatitis/eczema & minor dermatitis	Age, gender, atopy, ethnicity, indoor humidity, use of gloves and sanitizers	CIP ICD 51% IRR ICD daily freq. of handwashing: ≥ 10 vs < 10: **1.95 (**1.16–3.29) continuous variable: **1.04** (1.01–1.07) IRR Season (cold vs warm): **2.76 (**1.35–5.65)
Food-related industry					
Bauer et al. (1998) Germany	1 yr cohort study of apprentices at bakeries Interview and clinical exam 2–4 weeks after start of training *N* = 91, at ½ yr. *N* = 79 at 1 yr. *N* = 63	Self-rep freq. hr/day work tasks (< 1, 1–4, > 4) Work tasks incl. cleaning, wet dough, fruit handling	HE defined as mild (erythema, scaling), moderate (infiltration and papules), severe (vesicles and fissures)	Age, gender, atopy, previous HE, leisure activities	PP OCD / ICD hands: 2–4 weeks: 17.5% /NR, ½ yr: 29%/25.3%, 1 yr: 27%/19% HE, exp. OR Handwashing > 20/day: at 1 yr: 2.95 (0.85–10) Cleaning > 1 h: at 1 yr: **1.7** (1.3–2.1)
Bauer et al. (2001) Germany F-up Bauer et al. (1998)	3 yrs cohort study of Bauer et al. (1998) 63 (69%) at all 4 exam. after start of training	As Bauer et al. (1998)	As Bauer et al. (1998)	As Bauer et al. (1998)	PP 3-year OCD hands 27.5%, 21.7% ICD. Period P HE study: 41.3% Adj. analysis at 3-year, OR Wet work + Hand washing > 20 times/day: **1.2 (**1.05–4.77). Handwashing > 20 times/day, 1.2 NS
Hairdressers					
Uter et al. (1998a,b, 1999a,b) Germany	3-yr cohort study of hairdressing apprentices Ref: office workers *N* = 2352/111 at baseline 1-yr f-up: 1717/40 3-yr f-up: 1134/68	Self-rep daily time of wet work and of glove wearing High wet load: shampooing and permanent waving without gloves	Operational definitions for skin changes,classified Mild Moderate Severe	Atopy score, age, gender, atopy, past HE	PP any skin changes/HE %: 35.4/12.9 at baseline, 47.5/23.5 at 1 yr. f-up, 55.1/23.9 at 3 yr f-up Adj. analyses skin changes at 3 yr f-up, OR: Wet work /glove wearing hr/day < 2 h/ < 2 h: 1.4 (0.5–4.3), ≥ 2 h/ ≥ 2 h: **1.6 (**1.1–2.3), ≥ 2 h/ < 2 h: **1.8** (1.2–2.6), Handwashing > 10/day: 1.1 (0.9–1.4)
Wet work various industries					
Vermeulen et al. (2001) The Nederlands	CS study at nine rubber-manufacturing companies Interview and clinical exam *N* = 202 (90%)	Self-rep. freq. of handwashing, surfactants (mild soap or industrial) Use of gloves by observation Domestic exposure evaluated by experts for irritancy	Major dermatitis: erythema, papules, vesicles and fissures Minor dermatitis: erythema, chapping and scaling of the skin IgE for latex allergy	Age, gender, atopy, domestic skin exp. irritants	OR adj. *major/minor dermatitis:* Handwashing/day: 5– 9: 0.53 (0.11–2.66)/**3.09** (1.16–8.21) > 10: 1.18 (0.30–4.62)/2.27 (0.92–5.56) Industrial surfactant use: 0.64 (0.19–2.21)/1.92 (0.91–4.02) Glove use: 0.61 (0.18–2.11)/0.58 (0.27–1.23)
Gloves					
Weistenhofer et al. (2015) Germany	CS study in semiconductor manufacture company Interview and clinical exam Exp: clean-room workers Ref: admin personal *N* = 177/146	Nitrile gloves ≥ 2 h/day (whole work shift) Duration of exp: < 1, 1–2, 2–7.5, > 7.5 yrs	HE: clinical diagnosis using HEROS TWEL	Age, gender, atopy, smoking, use of barrier creams, skin care	Pos. ass. HEROS and duration of glove wearing, but no diff. between highest exp. and controls Adj. analyses: NS differences TEWL exp. vs controls: transient increased (30 min)
Weistenhofer et al. (2017) Germany	CS study, same population as Weistenhofer et al. (2015), new sample Interview and clinical exam Exp/ref: *N* = 270/ 135	Same exposure measurements as 2015 study 60% exposed < 7.5 yrs	As Weistenhofer et al. (2015)	As Weistenhofer et al. (2015)	Self-rep. skin problems at present workplace: 14% workers, 4% controls HEROS values: NS differences TEWL adj: increase TEWL ass. duration (yr) of employment in clean room
Metalworking/fluids and oils					
Berndt et al. (2000) Switzerland	2.5-yr. cohort study metalworker trainees Clinical exam. every 6-months *N* = 201 Nested CC of 47 cases	O-MWF, W-MWF, mechanical exp. metal dust, cleaning agents Duration individual exp. Based on expert judgement of job type and self-rep diary Schooldays without exp	Incident mild HE Case definition: erythema and scaling, vesicles, excoriations, papules or exudation	Age, gender, atopy, smoking, domestic exp	CIP 2.5 yr.: 23%. (*n* = 47), 9% in first ½ year OR multivariate analysis: Whole period/0.5 yr. prior to diagnosis: School days/week ≤ 1.5: **2.64** (1.23–5.67)/ **2.81** (1.20–6.56); Mechanical work (hr/day): 1.35 (0.98–1.86) trend *p* ≤ 0.1/ - Cleaners with solvent (hr/day:—/1.44 (0.99–2.08) trend *p* ≤ 0.1
Goh and Gan (1994) Singapore	6-month cohort study of machinist at ball-bearing factory and paramedic controls *N* = 24/27	O-MWF Qualitative exp. assessment of machinist with daily exp to cutting fluids	Point prevalence of ICD at 3 week intervals Clinical exam Mild: < 25% of hand, Moderate: > 25% of hand	Age, gender, atopy	PP ICD mainly mild at f-up week: 0: 0%, 3: 39%, 6: 78%, 9–30: ~50%. PP controls all 0
Mechanics—car industry					
Apfelbacher et al. (2010) Germany	Nested CC studies of irritant HE cases from 13- yr. f-up in car manufacture industry Controls: non-cases from same pop. Questionnaire, interview, clinical exam Cases/controls: *N* = 57/120	Self-rep. exp: Wet work > 2 h/day Dry skin soiling > 3 h/day W-MWF, O-MWF, solvents, epoxide, metal dust, cleaning/abrasive pastes	ICD by dermatologist HE when erythema and vesicles, scaling, papules, erosions/fissures, or lichenification	Age, gender, atopy, previous eczema, domestic exp	PP exp. ICD % / Controls %: Office job: 15.8% / 32.5%, **p = 0.02** Wet work ≥ 2 h/day: 49.1% /31.7%, **p = 0.02** Adj. analysis exp. ICD vs controls, OR: Office job: 0.61 (0.24–1.54) Wet work ≥ 2 h: 1.62 (0.78–3.37)
Various industries/exposures					
Chou et al. (2004) Taiwan, China	CS study of workers at a rayon factory Exp: *N* = 81 (CS_2_ *N* = 13; H_2_SO_4_ *N* = 2; combined * N* = 66) Ref: 29 packing, administration same company	Expert scoring by field study Exp. dichotomized ± CS_2:_ (undiluted) H_2_SO_4_ (20%) Combined: CS_2_ solution (2.2 g/l) & 10% H_2_SO_4_	HE Clinical diagnosed by dermatologist. HE: erythema, papules vesicles and fissures.	Age, gender handwashing habit & glove use given for 37 workers	PR HE: exp. 50–64%, ref. 3.4% HE Exp. vs ref. OR: CS_2_: **44.8** (6.4–934) H_2_SO_4_: **28.0** (0.8–1429) Combined:**49.0** (9.5–901)

Bold values indicate significant findings

Abbreviations: see footnote to Table 1

**Table 3:** Occupational exposures and self-reported outcome

Author Year Country	Study design and population	Exposure and exposure assessment	Outcome diagnostic criteria	Covariates accounted for	Results PR/RR/OR
Wet work occupations					
Cleaners					
Douwes et al. (2017) New Zealand	CS of cleaners and reference workers (retail and bus driving) *N* = 425/281	Job status at recruitment Self-rep. exp Self-rep. duration of daily exp. to water, cleaning products without gloves Use of gloves and skin care	HE or urticaria NOSQ-2002 questionnaire interview Prick test Measurement of TEWL	Age, gender, ethnicity, smoking, atopy Childhood eczema	Cleaners vs references: HE: 1.9 (1.1–3.2) HE due to glove use: 3.9 (2.2–6.7) Increased TEWL HE adj. analysis: Hours of hands exposed to water without gloves vs never, OR: < ½: **2.7** (1.2–6.4), ½–2: **3.3** (1.1–9.9); 2–5: 7.1 (1.7–29.8); > 5: **9.1** (1.5–56.2) No ass.to hand-washing, cleaning products or use of gloves
Mirabelli et al. (2012) Spain	CS study of cleaners and non-cleaners at 37 cleaning companies. Nested CC on subpopulation *N* = 693/125 (16%),	Self-rep: Freq. of use of various cleaning products Selected exp. last 12 months: Use of hydrochloric acid: 36%	HE last 12 months Definition HE: ≥ 1 of 5 possible self-rep skin symptoms Validation symptom-based HE: clinical exam. nested CC (70): sensitivity 0.82, specificity 0.62, pos. pred. 0.41, neg. pred. 0.92	Age, gender, previous eczema and allergy, frequent glove use, cleaning at home	Adj. PR Exp. vs C: **1.60** (1.03–2.47) Use of hydrochloric acid **1.92** (1.22–3.03) Use of dust mop products **1.75** (1.11–2.75) Also S for freq. use of several cleaning products
Nielsen (1996) Denmark	CS study of female cleaners at 271 public institutions *N* = 1166	Self-rep: Wet work hrs per week baseline: < 1:19%; 1–10:25%;11–20:33%; 21–30:16%; 31–40: 6%, > 40:1% Use of gloves	12-month prevalence of self-rep skin symptoms incl: red and rough skin; cracks, itching, vesicles No clinical validation	Age, gender, wet work at home Atopy not accounted for	Hr wet work and vesicles, OR: 1–10:1.4; 11–20:1.6; 21–30:**2.4;** 31–40:2.0; > 40:0.9 Use of protective gloves and vesicles, OR Seldom: 1; sometimes **3.1**, often **4.2**
Healthcare workers					
Hamnerius et al. (2018) Sweden	CS study 12.228 hospital employees, 9051 HCW (nurses, assistant nurses and physicians)	Self-rep. % exp, times a day: Handwashing with soap 30% > 20 times Alcoholic Hand disinfectants: 45% > 50 times Gloves: 40% > 20 times Hours a day: Gloves, 54% > 2 h	HE 1-yr and life time prevalence	Age, gender, AD wet work at home life style,	1 yr. prevalence HE: 21% Life time prevalence HE: 35% Adj. OR: Times/day hand wash with soap: 11–20: **1.3** (1.1–1.6); > 20: **1.4** (1.1–1.8) Hours of daily glove use: 1–3 h/ day: 1.2 (0.97–1.5) > 3 h/day: **1.5** (1.1–1.8)
Visser et al. (2014b) The Netherlands	1–3 yrs. prospective cohort study of apprentice nurses from 15 vocational schools 721 (50% baseline), 533 (73%), f-up 1,2 or 3 yrs., 445 without previous HE	Diaries. Median freq./day: Handwashing: 8 Alcoholic hand disinfection: 5 Gloves: 4 Other soap/detergents: 4 Other disinfectants: 2	HE: self-rep. symptoms > 3 days No clinical validation	Age, gender, Higher proportion of atopics among participants than non-participants No adjust AD	IR HE/100 person-yr in traineeship 36.7 first vs 13.7 s/third Wet work activities, adj. OR: Handwashing ≥ 8 times/day: **1.5** (1.02–2.25) Other Soap exp. ≥ 4 times/day: 1.5 (0.97–2.30) Disinfectants (non-alcoholic) ≥ 2 times a day: 1.1 (0.69–1.79)
Visser et al. (2014a) The Netherlands	As (Visser et al. 2014b), same study population 626 DNA samples, 596 genotyped for four filaggrin gene mutations	As (Visser et al. 2014b) Self-rep. work in a side job (i.e., healthcare, catering)	HE: as (Visser et al. 2014b)	Atopy, FLG-mutations, freq. handwashing at home	Adj. analysis HE, OR: Handwashing ≥ 8 times/day: **2.2** (1.2–4.2) Side job wet work > 8 h./week: **1.8 (***1.1–2.9)*
Lee et al. (2013) Korea	CS study among nurses Questionnaire. Patch testing on subpopulation *N* = 525	Self-rep. daily freq handwashing, gloves and hand moisturizer Gloves min per use, 21% > 5 min	HE: last 12 months Symptom-based HE ACD: relevant pos. patch tests in 43 (61.4%) of subgroup of 70 (43%) of workers with HE	Age, gender, atopy, history of rhinitis/asthma. hours of housework	HE: Adj. OR’s: Handwashing times/day: 10–19: 1.31 (0.71–2.36); 20–29: **5.77 (**2.53–13.2); > 30: **13.1** (3.48–49.2) Glove wearing min per use 1–5: 1.6 (0.96–2.65); > 5: **1.99** (1.01–49.2) Use of moisturizer times a day Significant prevention
Ibler et al. (2012) Denmark	CS study of HCW (physicians, nurses, nursing assistants and clinical assistants) *N* = 2269	Self-rep. exp: Handwashing, hand disinfectants, water exp, gloves, detergents	HE: last 12 months NOSQ-2002 questionnaire	Age, gender, domestic exp	21% HE last 12 months HE freq hand washes/day vs no hand wash, OR* 1–5: **0.35** (0.12–0.99); 6–10: 0.39 (0.14–1.10); 11–15:0.52 (0.18–1.48); 16–20: 0.74 (0.26–2.10) No ass.to hand disinfectants, duration of daily water exp., detergents or glove exp
Lan et al. (2011) Taiwan	Study 1 CS study of nurses from university hospital *N* = 1132	Work experience, 32% > 10 yrs Work section: outpatient clinic, ward, special care unit	HE based on diagnostic algorithm of symptoms Atopic eczema during the past year	Age, gender, atopy, housework	PP HE: Yrs. work experience: < 5: 18.7%, 1; 5–10: 20.3%, 1.03 (0.71–1.50); > 10: 27.3%, Adj. OR **1.52** (1.07–2.17) Work section NS
	Study 2 Observational study among 140 non-atopic nurses with > 1 yr. experience participating in study 1	Observations during 4 h morning shift, times per day and daily duration of Handwashing Alcohol hand rub Gloves:	As above	As above	Adj. OR HE: Handwashing > 6 times: **3.02** (1.26–7.23) Alcohol rub > 9 times: 0.62 (0.26–1.50) Gloves > 2 times: 0.51 (0.17–1.48) Daily duration of exp. NS
Flyvholm et al. (2007) Denmark	CS study of hospital employees. Questionnaire *N* = 1246	Self-rep: Jobs Daily hand-washing, > 20 times: 44% Daily or weekly use of hand disinfectants 54.3% Use of gloves: 46.8%	HE last 12 months NOSQ-2002 questionnaire	Age, gender, AD, rhinitis, asthma HE more frequent among atopic, females, < 40 yrs	PP HE in job groups varied 7.9%–32.1%, PR* HE: Handwashing > 20 times daily: 1.83*(1.47–2.28); Hand Disinfectants daily/weekly: 1.07* (0.85–1.34); Use of gloves: 1.87* (1.43–2.43)
Hairdressers					
Jung et al. (2014) Korea	CS study of random sample hairdressers. Questionnaire *N* = 1,054	Self-rep. exposure: Training status Washing, cutting, permanent wave or dying Hairdressing chemicals Personal protective equipment (gloves, mask)	HE: self-rep. symptoms > 3 weeks	Age, gender, smoking, alcohol, perceived health, personal protective equipment	Adj. OR HE: Training status vs master: designer 1.22 NS, staff **2.70** (1.32–5.51) Washing vs cutting: **2.03 (**1.22–3.37), Exp. to chemicals 0.89 (0.53–1.49)
Wet work various industries					
Mortz et al. (2014) Denmark	Cohort study of young adults, age 28–30 yr from the general population (15 yr. after baseline study) Questionnaire * N* = 899 (75% of available at f-up) Clinical exam. * N* = 469	Self-rep. exp. wet work Handwashing > 20/day: 10.7% Use of occlusive gloves Domestic exp	HE (current, 1 yr, lifetime) NOSQ-2002 questionnaire Subgroup clinical HE Patch test incl	Age, gender, atopy, smoking, domestic exp	Current HE 7.1%, 1 yr PP. 14.3%. Clinical HE 6.4% (76% ICD, 3% ACD) HE self-rep 1 yr: Handwashing > 20 vs 0–5 times/day, OR*: **2.48** (1.38–1.45). DRR* to freq of handwashing, **p** = 0.01 Adj. analyses (OR) current wet work **1.7** (1.1–2.8)
Lazarov et al. (2005) Israel	CS study among 400 hydrotherapist Interview and questionnaire *N* = 190	Cumulative work hrs in pools. 20% > 10,000 h 59% present hydrotherapist	Self-rep symptoms: extremities, face and trunk No clinical validation	Age, gender AD, smoking Pre-existing skin disease (11.9%),	PP self-rep. skin disease 45% (*n* = 85) Adj. analysis, OR ≥ 10,000 h cumulative exp: **2.81** (1.07–7.37)
Metalworking/fluids and oils					
Mirabelli et al. (2009) 10 European Countries	Pooled data from two multicenter population-based f-up studies Baseline surveys: 1991 F-up: 1998/2002 *N* = 676	Self-rep. days of weekly exp Metalwork W-MWF, O-MWF Organic solvents/degreasing agents	Self-rep rash during last 12 months No distinction between irritant and allergic reactions	Age, gender, atopy, history of eczema or skin allergy at baseline	12-month PP 10% Days of weekly exp. 1–3; 4–7. Ref < 1 day. Adj. PR Hard Metal: **1.52** (1.07–2.16),1.86 (0.96–3.62) O– MWF: 0.89 (0.44–1.81),**1.16** (1.25–2.49); Organic solvents: **1.84 (**1.14–2.97), **2.06** (1.21–3.50)
Construction/cement					
Avnstorp (1991) Denmark	6-yr cohort study construction workers exp. to cement, not sensitized to chromate *n* = 67 (46%)	Self-rep. freq. work processes involving cement exp Use of gloves, creams and handwashing	ICD: Self-rep. episodes of HE previously/past/current lasting at least 2 weeks Neg patch test to chromate	Allergy chromate	CIP self-rep. HE 16.4% NS ass. HE and concrete pouring and other work task with cement exp
Petersen and Sabroe (1991) Denmark	CS study of construction workers exp. to mineral wool (MMMF) Questionnaire *N* = 2654	Self-rep. MMMF exp. hr per month past yr	Self-rep eczema when skin eruption/rash > once per week Self-rep. stinging or itching	Age, gender, smoking, organic solvents	2/3 of highest exp. had symptoms from skin **DRR** in adj. analysis OR symptom each of 6 exp. categories: Itching skin: **1.48 (**1,39–1,55) Eczema: **1.29 (**1.21–1.38)
Daftarian et al. (2002) USA	CS study at a foam manufacturing facility exp. to toluene diisocyanate (TDI) and controls of non-production workers *N* = 88/26	TDI and other chemicals (waxes and adhesives) TDI measurements foam production: Individual measurements in breathing zone, area samples, ± dermal exp	Irritant reaction based on self-rep. dermal symptoms, no specific antibodies (IgE and IgG) and neg patch test to TDI	Age, gender, sensibilisation environmental allergens (IgE)	Production vs non-production workers: PR: 2.66 (1.14–16.32) No type 1 or type 4 allergy to TDI Work-related dermal symptoms was irritant reaction to TDI

Bold values indicate significant findings

Abbreviations: see footnote Table 1

**Table 4:** Prognosis of ICD

Author Year Country	Study design and population *N* (% participation)	Exposure and occupational consequences	Prognostic outcome diagnostic criteria	Covariates accounted for	Results PR/RR/OR
Caroe et al. (2018b) and sub-study (Caroe et al. 2018c) Denmark	Cohort study of pt. with recognized OCD 4–5 yr. after diagnosis OCD *N* = 1,496 (58% of baseline) ICD * N* = 1,067 ICD wet work: * N* = 954	ICD due to wet work: 43% HCW Job change during follow-up of ICD: Same profession: 50% Changed profession: 31% Not working: 18% Duration of wet hands and glove use, freq of handwashing at f-up	Healing during 12 months and improvement in severity Severity scale 0–10, of current OCD Effect of job change on quality of life	Age, gender, AD Sub-study: severity ICD at baseline	*Healed* ICD/OCD and profession, OR: a) Changed vs same prof: 1.60 (0.99–2.61) / **1.62** (1.06–2.47) b) Not working vs same prof: **2.68** (1.59–4.60)/ **2.85** (1.83–4.24) *Improved* ICD wet work and profession, OR: a) **2.13** (1.49–3.05) b) **1.79** (1.19–2.70)
Vester et al. (2012) Denmark	Cohort study among food-related jobs 0.3–10 yr *N* = 178 (69%) Protein CD: 28% (50) ICD: 63.5% (113) ACD, CU, multiple: 8.5% (15)	Work-related consequences Job change due to skin problems ICD ~ others: 43% Sick leave > 3 weeks: ICD ~ others:10%	Improvement in subgroups of OCD of hands At baseline clinical diagnoses of subgroups of HE, incl. ICD	Age, gender, AD	84% *improved* after job change with no difference between diagnostic groups comparing protein CD with others (ICD 88%)
Malkonen et al. (2009) Finland	Cohort study of patients diagnosed with OSD incl. ICD. Questionnaire 6 month f-up OSD 1048 (89%) Subgroup with ICD: 363	ICD and changed work tasks: 19% job/occupation: 12% loss of job: 13% ICD, no work changes: 47%	Continuation or healing of ICD, diagnosed clinically at baseline, incl. patch and prick test	Age, gender, atopy, contact allergies, occupation	*Healed* ICD: 23% *Continuous* OCD, OR: ACD ref. ICD: 1.0 No work changes: **2.7** (1.9–3.8), Food related occupation**: 1.8** (1.1–3.1) Skin atopy: 1.4 NS Respiratory atopy: **1.8** (1.1–3.1) Age > 45 yr: **2.3** (1.7–3.3)
Malkonen et al. (2010) Finland	Study population as Malkonen et al. (2009). Questionnaire-based f-up 7–14 yr (mean 10.5) *N* = 605 (80.1% OCD) ACD: *n* = 354 ICD: *n* = 251	ICD, work changes work tasks or job: 55% occupation: 35% loss of job: 18% no work changes: 8%	Self-rep healing of baseline OCD	Age, gender, skin atopy, work-related contact allergy	*Healed* OCD*,* ACD & ICD alike *Continuous* OCD, OR Duration of OCD prior to diagnosis (ref < 1 yr) 1–2 yr: **3.1** (1.8–5.2); 2–5 yr: **1.9** (1.2–3.1); 5–10 yr: **2.6** (1.4–4.6); > 10 yr: **4.6** (2.4–8.7) No change of occupation **1.6** (1.03–2.34) Skin atopy: **1.9** (1.1–3.2) Respiratory atopy: **2.7** (1.4–4.9)
Cvetkovski et al. (2006) Denmark	Cohort study pt. with recognized OCD—1 yr. f-up *N* = 564 (91%) ICD: 61% ICD + ACD or CU: 15%	Job change at f-up: 48% Disease duration Socioeconomic status	Aggravation, persistence or improvement of self-rep. severity of HE: visual analog scale At baseline, clinical scoring of severity by dermatologist	Age, gender, atopy, occupation	I*mproved* HE 41% *Aggravated or persisting* HE (RR): AD: **1.53** (1.1–2.2) Age > 25, worse prognosis No ass. diagnostic group Risk of job loss severe OCD RR **14.0**
Jungbauer et al. (2004b) The Netherlands	Cohort study of patients diagnosed with ICD by dermatologist F-up 5 yr N = 124 (72%)	Wet work exp. at f-up Low: 48%, Medium: 9% High: 42% Self-rep. preventive measures	Self-rep. disease severity Freq. of visiting dermatologist	Age, gender No ACD or AD at baseline	Severity scores 5 yr. after diagnosis: medium 50%, high in 32% No significant association between severity of HE and parameters for exp, gender or occupation
Adisesh et al. (2002) UK	Cohort study of consequences of reported OCD. Questionnaire and f-up after ½ and 1 yr N = 510 (71%) 1/3: ICD, 1/3 ACD, 1/3 mixed	Exp. duration < ½ year. 17% ½-3 yr. 22% 3–10 yr. 27% > 10 yr. 18% Unknown: 17%	Time off work Improved clinical condition during f-up OCD diagnosed clinically Patch test of 61%	Age, gender, atopy	Time off work, OR ACD vs ICD/mixed: **1.8** (1.1–2.8) Age/per 10 yr: **1.3** (1.1–1.5) Notified OCD: **4.4** (2.2–8.9) *Improved* OCD 84.3% Mean duration of exp. non-improved vs improved Non-atopic: 9.1 yr. vs 5.3 yr. (p = 0.03)
Shah et al. (1996) UK	Cohort study of self-rep. prognosis of OCD in metalworkers 1–5 yr. after clinical diagnosis OHE: 51 (80%) ICD: 31% (16), ACD: 53% (27), AD 16% (8)	Exp. cutting oil f-up: 43% continuous exp 55% of non-exposed/ unemployed or retired	Self-rep. continuous symptoms of HE Baseline clinical diagnoses: ACD: pos. patch test ICD: Irritant factors evaluated as important	AD incl. as subgroup of OHE	*Continuous symptoms* PP 82%, no S difference diagnoses Continuous exp. vs non-exposed *Continuous symptoms*: 86% vs 79%, NS
Rosen and Freeman (1993) Australia	Cohort study of patients with OCD F-up: 1–5 year OCD: 334 (59%) ICD: 58% (195) ACD: 42% (139)	Self-rep. changes Changed industry: 37% (n = 122) Hairdressers: 47% Food industry: 39% Changed work tasks: 24%	Self-rep. status of healing and improvement i 5 outcome categories Baseline clinical diagnosis incl. patch test	Atopy	Improvement rate 70% Change of occupation vs same industry *Healed*: 43% vs 28%; RR*1.6 (1.2–2.1) *Improved:* 76% vs 67%, RR* 1.14 (1.0–1.3) Change of work tasks vs same work tasks: *Healed:* 43% vs 24%; RR*1.8 (1.2–2.8) *Improved:* 82% vs 61%, RR*1.4 (1.1–1.6)
Chia (1991) Singapore	Cohort study of patients with OCD. F-up 1 yr N = 112 (87%) ACD 28% (n = 31) ICD 73% (n = 81)	Prognostic factors: ICD duration exp. yrs: < 1: 43%,1- < 3: 31%; ≥ 3: 26% Prevention: avoiding exp: Oil/coolants: 46% (11) Solvents/flux: 52% (12)	Healing or persistence of OCE, Clinical assessed in 41%, interview- based in 59% Baseline clinical diagnoses, incl. patch test	Age, gender, ethnicity, ACD	OCD *healed* in 72% of cases No difference between ACD and ICD ICD, in stratified analyses: Prognosis did not depend on age, sex, ethnicity, duration of exp or avoidance of exp
Lindemayr (1984) Austria La. German	Cohort study of hairdressers with OCD Observations: 0.3–2 yr OCD N = 215 (87%) ACD 71% (n = 154) ICD/non-ACD 29% (n = 61)	Change of occupation: 62% Detailed listing of types of chemicals used at baseline	Healing of OCD, assessed clinically Baseline clinical diagnosis incl. patch test	Gender, atopy	*Continoued* occupation ACD: 32% *Healed,* ICD 58% *Healed* *Discontinue*d occupation *ACD: 60.4%* Healed*, ICD: 67.5%* Healed
Keczkes et al. (1983) UK	Cohort study of patients with ICD. F-up 1–16 year N = 188 (41%)	Change of occupation: 49% 24% were housewives/part-time cleaners	Self-rep. healing or active ICD ICD diagnosed clinically at baseline, all neg. patch tests	Exclusion of patients with history of AD or psoriasis	PP (95% CI*): *Healed* in 31% (24–38) *Healed* ICD when change of occupation vs same occupation PP 33% vs 30%, PR* 1.10 (0.7–1.7)

Bold values indicate significant findings

Abbreviations: see footnote to Table 1

Table S3 in the online supplementary material presents summary of the quality assessment of the selected papers.

## Wet work: exposure to water, disinfectants, and detergents/soaps

### Design

Exposure to disinfectants and soaps/detergents may itself cause irritation, although these exposures are often reported in combination with wet work and, therefore, the effect may be difficult to differentiate.

The main occupations recording wet work exposure in this review are studies conducted among healthcare workers (HCW), cleaners, hairdressers, and various industries like the food-related industry and manufacturing of rubber.

Exposure to wet work was reported in 21 epidemiological studies, 7 were follow-up studies, 12 cross-sectional studies, and 1 was a nested case–control study in specific occupations and industries (Tables 1, 2, 3). Furthermore, a population-based follow-up study of young adults from the general population was included.

### Exposure source and measure

A study by Lan et al. (2011) among non-atopic nurses applied quantitative measurements of wet work exposure by observation, along with measurements of exposure to disinfectants and glove use. A follow-up study among apprentice nurses used diary cards to provide semi-quantitative measurements (Visser et al. 2014b).

The remaining studies relied on self-reported information on exposures in questionnaire and/or interviews.

Some of the studies included other exposure variables, such as fruit preparation and cleansing (Bauer et al. 1998), contact with specific detergents (Teo et al. 2009) and disinfectants (Flyvholm et al. 2007; Hamnerius et al. 2018; Held et al. 2001; Visser et al. 2014b), or industrial surfactant (Vermeulen et al. 2001). Studies of use of gloves, which may be a part of wet work, but will be treated separately in this review.

### Outcome

Diagnosis of outcome was clinically assessed in nine studies (Apfelbacher et al. 2010; Bauer et al. 1998; Callahan et al. 2013; Guo et al. 1994; Held et al. 2001; Stingeni et al. 1995; Teo et al. 2009; Uter et al. 1999a; Vermeulen et al. 2001). Typical categories were mild or minor dermatitis, with irritant reactions described as slight erythema, chapping, and scaling, without morphology of papules, vesicles or fissures, and moderate and severe or major dermatitis.

In six of the studies without a clinically verified diagnosis, outcome relied on self-reported dermal symptoms resembling HE (Jung et al. 2014; Lan et al. 2011; Lee et al. 2013; Mirabelli et al. 2012; Nielsen 1996; Visser et al. 2014b), and in one study, the dermal symptoms affected other body parts than the upper extremities (Lazarov et al. 2005). In a study among nurses, the questionnaire was supplemented by a patch test in a sub-cohort (Lee et al. 2013). Five studies relied on self-reported HE (Hamnerius et al. (2018)) and four based on the same standardized questionnaire (Douwes et al. 2017; Flyvholm et al. 2007; Ibler et al. 2012; Mortz et al. 2014).

### Quality of the studies

All the included studies could potentially be affected by bias or confounding. In the cross-sectional studies, exposure and outcome were collected simultaneously and a causal relation can only be suggestive. Selection bias due to healthy worker effect is likely in most studies and may attenuate the results of these studies, with a probable direction of bias toward unity. This is also the case if more susceptible individuals, i.e., individuals with atopic skin diathesis, are less likely to be exposed due to pre-work self-selection (Uter et al. 1999b).

As most of the studies relied on self-reported qualitative exposure assessment, it is also probable that exposure misclassification is present, leading to non-differential misclassification with dilution of exposure contrast, and possible attenuation of results.

In addition, studies may have been affected by a misclassification of outcome, particularly studies with questionnaire-based outcome. This is especially the case with studies with a symptom-based outcome in which there is a high prevalence of symptoms and low specificity, but if the outcome is non-differential, this will cause bias toward the null.

It is not possible to distinguish between ACD and ICD without including patch tests, and while wet work especially hand-washing can be regarded as a likely irritant, the lack of clinical assesment of possible ACD including patch testing for allergens is a major limitation in these studies.

Taken together, all the studies had flaws of minor or major character, and no studies were regarded as being of the highest quality. Three studies were graded as high quality (Callahan et al. 2013; Lan et al. 2011; Uter et al. 1999a), 11 as medium quality, and the remaining 7 studies as low quality (for details, see online supplementary table S3).

### Results

Overall, the studies across industries consistently pointed toward a moderate or low association between wet water exposure and probable ICD, especially regarding the *frequency of exposure.* We found no effect of alcoholic hand disinfection.

Callahan et al. (2013) in a 6-month follow-up study of HCW in the USA reported a dose–response relation to clinically verified HE for handwashing frequency as a continuous variable for both the adjusted point prevalence rate and the incidence rate ratio (IRR) of approximately 1.04, and also to handwashing frequency ≥ 10 times a day; point prevalence rate 1.55 (1.01–2.39), and IRR 1.95 (1.16–3.29) (Table 2).

Ibler et al. (2012) in a study on Danish HCW reported an increase of HE across five categories of handwashing until > 20 hand washes.

Lee et al. (2013) in a study of Korean nurses reported a uniform increase in OR for self-reported HE across four handwashing categories, with a reference of < 10 hand daily hand washes, significant for the categories 20–29 times/day [OR 5.8 (2.5–13.2)] and for > 30 times/day [OR 13.1 (3.5–49.2)] (Table 3).

Hamnerius et al. (2018) in a large cross-sectional study of about 9000 Swedish HCW reported a significant dose–response relation for HE for handwashing with soap, with OR 1.3 and 1.4 for 11–20 and for > 20 times/day, respectively (Table 3).

Among HCW, Visser et al. (2014b) in a follow-up study of apprentice nurses reported non-significant associations between HE and use of both soaps and non-alcholic disinfectants, but no associations with use of alcoholic hand rubs. Held et al. (2001) in a intervention study of apprentice nurses reported aggravation of skin problems associated with use of hand disinfectants (non-specific), OR 6, and Stingeni et al. (1995) in a study of hospital employees reported disinfectants, mainly chrohexidine gluconate and glutaraldehyde, and detergents/soaps,to be the main causes of OICD, while the frequency related to alcohol-based disinfectants was low. Other studies on HCW have reported no association between HE and use of alcoholic hand disinfectants (Hamnerius et al. 2018; Lan et al. 2011; Lee et al. 2013), or non-specified hand disinfectants/local disinfectans (Flyvholm et al. 2007; Ibler et al. 2012).

Vermeulen et al. (2001) in a study of rubber-manufacturing workers reported adjusted OR of minor dermatitis significantly increased for handwashing 5–10 times/day of 3.1 (1.2–8.7), but not for > 10 times/day, although the latter also gave an OR above unity of 2.3 (0.9–5.6). When combined with the use of industrial surfactant, but not regular hand soap, they found a dose–response relation with an OR of 4.3 (0.9–20.3) and 6.4 (1.4–30.7) for handwashing 5–9 and > 10 times per day, respective (Table 2).

Several cross-sectional studies have reported increased prevalence ratios or OR for dichotomized handwashing frequencies ranging from > 8 times/day to > 20 times/day and ratios in most studies ranging from 1.8–3.0 (Tables 1, 2, 3). Two follow-up studies reported non-significant ratios at follow-up for handwashing > 10 times/day (Uter et al. 1999a) and > 20 times/day (Bauer et al. 1998) (Table 2).

The *duration of daily wet work* has less consistently been associated to ICD. Uter et al. (1999b) in a follow-up study of German hairdressing apprentices reported especially unprotected wet work > 2 h per day to be significantly associated with HE, with OR increasing from 1.6 when using gloves to 1.8 without gloves. Douwes et al. (2017) in a study of cleaners also reported a dose–response relation between self-reported HE and duration of daily exposure to water without gloves. Similar significant and insignificant trends suggestive of a dose–response effect have been shown in a number of cross-sectional or follow-up studies in cleaning and other industries in which handwashing has been registered as number of hours per day or week (Tables 2, 3).

In contrast among HCW, Lan et al. (2011) in an observational study of exposure and Ibler et al. (2012) in their study reported no association of HE to duration of daily handwashing.

### Conclusion

The available evidence from epidemiological studies supports an association between wet work, especially frequent wet work and mostly minor ICD. No threshold level can be described. The level of evidence is considered strong (+++). Results from the presented studies of exposure restricted to disinfectants and detergents vary. Evidence from combined exposure to water, probably the case for multiple studies on wet work exposure where these exposures cannot be separated, indicates detergents especially industrial surfactants and non-alcoholic disinfectants as an important cause of ICD. The overall evidence of a causal association between ICD and exposure to detergent and disinfectants is considered moderate (++), while the overall evidence combined with other wet work is considered strong (+++).

## Exposure to gloves

### Design

Exposure to gloves and outcomes related to ICD were reported in 14 epidemiological studies, including 10 studies also described in the section concerning wet work (Tables 1, 2, 3). Occupations included studies in HCW (8), cleaning (2), hairdresser apprentices (1), workers in a rubber-manufacturing plant (1), and clean-room workers in a semiconductor production company (2). One study was a follow-up study (Uter et al. 1999a), the remaining cross-sectional studies.

### Exposure source and measurement

In one study, exposure assessment to gloves was based on observations (Lan et al. 2011) and in another study recorded on diary cards (Visser et al. 2014b). In studies of clean-room workers, all workers were considered exposed to occlusive gloves during all work hours (Weistenhofer et al. 2015, 2017). The remaining studies relied on self-reported exposure of number of hours per day.

### Outcome

Six studies included clinical verified diagnosis, two of which also included patch tests (Stingeni et al. 1995, 1996). In five studies, diagnosis was self-reported HE in the past 3 or 12 months. Three studies relied on self-reported symptoms (Table 3).

### Quality of the studies

Possible selection bias in the cross-sectional studies reporting adverse effects of glove wearing is a major risk factor, as a reverse causation cannot be ruled out.

Use of gloves, especially rubber gloves, is also a risk factor for ACD and use of natural rubber gloves a risk factor for CU. For general methodological issues, we refer to this section in the paragraph on wet exposure.

Overall, four studies are graded as high quality (Lan et al. 2011; Uter et al. 1999a; Weistenhofer et al. 2017, 2015), five medium quality (Douwes et al. 2017; Hamnerius et al. 2018; Lee et al. 2013; Vermeulen et al. 2001; Visser et al. 2014b), and the remaining low quality.

### Results

Stingeni et al. (1995) reported results from two studies of employees from the same hospital who were clinically examined including patch tests. In the first study, they found a high frequency of OCD (21%) and IOCD in 95% of cases (Stingeni et al. 1995). In the second study, which only included workers using latex gloves, ICD was diagnosed in 13%, by a positive “user test”. After testing with two types of latex gloves, they found 36% of cases to be associated with corn starch powder, and 28% with corn starch and/or latex-protein (Stingeni et al. 1996). Hamnerius et al. (2018) in a Swedish study examined self-reported HE among more than 9000 HCW. They found a dose-dependent association between HE and the duration of daily glove use: OR 1.5 (1.1–1.8) for the highest exposure category of more than 3 h/day, but no association with frequency of glove use.

Three studies on cleaners (Nielsen 1996) and HCW (Flyvholm et al. 2007; Lee et al. 2013) reported positive associations between use of gloves and increased risk of self-reported symptoms of HE, corresponding to an increased OR of 1.87 for self-reported HE with use of protective gloves (Flyvholm et al. 2007) and of 1.99 for wearing of gloves more than 5 min per use but with no association with frequency of glove use (Lee et al. 2013). By contrast, no increased risk for ICD was reported in six studies (Douwes et al. 2017; Uter et al. 1999a; Vermeulen et al. 2001; Visser et al. 2014b; Weistenhofer et al. 2015, 2017).

Uter et al. (1999a) in a follow-up-study on hairdressing apprentices reported a protective effect on HE with use of gloves for more than 2 h per day.

Weistenhofer et al. (2017); (2015) performed two studies, 1 year apart, at the same company in 177 and 277 clean-room workers using occlusive nitrile gloves for most of their work shift, compared to reference workers with no glove exposure. In both studies, they reported that clean-room workers had an increased frequency of self-reported work-related skin problems, but no difference in clinical hand eczema score (HEROS).

Vermeulen et al. (2001) found no association between glove use and clinical diagnosed HE or minor dermatitis among rubber-manufacturing workers. Neither did four studies on self-reported HE among HCW (Ibler et al. 2012; Lan et al. 2011; Visser et al. 2014b) or cleaners (Douwes et al. 2017), which in the study by Lan et al. (2011) included observations of glove use.

### Conclusion

The studies on glove exposure vary with only slightly increased ICD in the studies of highest quality. While some studies have mainly shown positive effects of glove use in relation to HE, other studies have revealed associations between HE/ICD and daily use of gloves, i.e., a clinically negative effect of gloves use.

The overall evidence of a causal association between ICD and occlusive glove exposure without other irritant exposures is considered limited (+).

The overall evidence of a causal association between ICD and occlusive gloves combined with other irritant exposure is considered moderate (++).

## Metals, metalworking fluids, and oils

### Design

Exposure to MWF and outcomes related to ICD were reported in seven epidemiological studies, one follow-up study, three nested case–control studies within follow-up studies, and three cross-sectional studies.

Five studies were from different kinds of metalworking factories and one from a car manufacturing industry. The sevent study was a case–control study within a population cohort.

### Exposure, source, and measure

Two studies used an external control group, and the remaining relied on exposure contrast within the exposed groups, based on expert evaluation of individual job risk factors with or without work diaries (Berndt et al. 2000; Jee et al. 1986). In a large study in a hard-metal production facility, the exposure to cutting oils and fluids relied on observations on work tasks on the day of examinations (Fischer and Rystedt 1985). Two studies relied on self-reported exposure intensity (Apfelbacher et al. 2010; Mirabelli et al. 2009).

Five studies reported exposure to MWF, oil-based MWF (Goh and Gan 1994), combined with water-based MWF (Berndt et al. 2000; de Boer et al. 1989), and with mechanical exposures representing mechanical friction (Berndt et al. 2000; Mirabelli et al. 2009).

### Outcome

Diagnosis of outcome was clinically assessed in all but one study and included patch test in three studies and all provided some information on criteria for diagnosis or grading of diagnosis. Three studies included varying degrees of minor irritant reactions, including typically slight erythema, dryness, and chapping. One study used the definition “clinical dermatoses” combining minor changes and eczema; three reported on clinically ICD. In one study, outcome relied solely on self-reported symptoms of a rash (Mirabelli et al. 2009).

### Quality of the studies

General methodological issues regarding risk of selction bias, miclassification of outcome, and exposure were election bias applied as in the section on exposure to wet work.

Overall one study was regarded as high quality (Berndt et al. 2000), three studies as medium quality, and two as low quality (Fischer and Rystedt 1985; Goh and Gan 1994).

### Results

Exposure to kerosene was only reported in one study of medium quality. In this study, there was a very high prevalence of clinical dermatoses including (84% of the workers), with a a prevalence ratio of 5.5 compared to reference workers, while no differences suggesting a dose–response relation could be found when comparing high exposed with low exposed (Jee et al. 1986).

One large 2.5-year prospective study of Swiss metalworker trainees in a nested case–control design reported associations between lack of rest days, mechanical work, and exposure to cleaning agents containing solvents, while there was no separate effect of exposure to MWF or metal dust (Berndt et al. 2000).

Another nested case–control study in the car industry could not demonstrate any significant effect of exposure to metal-related work exposures (Apfelbacher et al. 2010).

The remaining three studies reported increased risk of generally mild HE, with prevalence ratios ranging from 1.2 to 3.7 (de Boer et al. 1989; Fischer and Rystedt 1985), and in one study point prevalences up to 78% were found in exposed compared to none in reference workers (Goh and Gan 1994).

No studies reported on dose–response relation between exposure to MWF and irritant skin changes.

### Conclusion

The available evidence from epidemiological studies supports a moderate level of association (++), between MWF and mainly minor ICD.

## Mechanical exposures

### Design

Mechanical exposure related to irritant skin reactions was reported in four industry-based, one nested case–control study within a follow-up study and three cross-sectional studies (Tables 1, 2, 3). Two of the studies were from the metalworking industry and have also been included in the section on MWF and two were from the construction industry involving exposure to airborne man-made mineral fibers (MMMF).

### Exposure, source, and measurement

Measurements of dust exposure with diameters of the ceramic fibers were performed in one study (Kiec-Swierczynska and Wojtczak 2000). In the other construction study, exposure to MMMF was self-reported (Petersen and Sabroe 1991). One study by Berndt et al. (2000) included expert-based semi-quantitative exposure assessments, while Fischer and Rystedt (1985) collected present exposure by observation.

### Outcome

Diagnosis of ICD and irritant reactions were based on clinical examinations in three studies, including patch test in two studies, and self-reported eczema and symptoms in the fourth study. All studies provided some information on criteria for diagnosis of ICD/HE and/or irritant symptoms.

### Quality of the studies

The three cross-sectional studies are prone to selection bias. Misclassification of both exposure and outcome was most likely in the study by Petersen and Sabroe (1991), which relied solely on self-reported data, and in the study by Fischer and Rystedt (1985) which did not distinguish mechanical exposure of metal and powders from cutting fluids. Overall, two of the studies was evaluated as being of high quality (Berndt et al. 2000; Kiec-Swierczynska and Wojtczak 2000); one of medium quality and one of low quality.

### Results

Kiec-Swierczynska and Wojtczak (2000) found increased prevalence of both acute and chronic ICD among workers exposed to MMMF, corresponding to an overall prevalence ratio for ICD of 6.9* (2.3–20.8) compared to non-exposed, and a patch test with the ceramic fibers confirming irritancy of the fibers. Petersen and Sabroe (1991) reported a dose–response relation between exposure to MMMF and self-reported eczema and itching of the skin.

In the studies of metalworkers, the nested case–control study reported a non-significant trend for a dose–response association between hours of daily mechanical work and incident cases of mild HE (Berndt et al. 2000), while the cross-sectional study described an increased prevalence of irritant reactions, prevalence ratio 3.7* (2.7–4.9) (Fischer and Rystedt 1985).

### Conclusion

The reported epidemiological documentation for ICD and skin irritation due to mechanical irritation among workers is scarce. The few published studies do not allow for a firm conclusion, and the evidence of a causal association is therefore limited (+).

## Prognosis of ICD

### Design

Epidemiological studies on prognosis of OCD including OICD, with focus on healing or improvement, were reported in 13 papers from 10 prospective studies and one retrospective cohort study (Table 4).

The studies were based on follow-up of clinically diagnosed cases by dermatologist and/or occupational physician (Adisesh et al. 2002) or nationally notified recognized cases.

Eight studies concerned patients from a broad specter of various industries. Three studies focused on workers from specific industries: the food industry, a metal processing plant, and hairdressers.

The follow-up time in the studies ranged from 0.3 to 16 years, but most had fairly short follow-up periods of ½-1 year and up to 5 years.

### Exposures and occupational variables influencing prognosis

The main outcome of interest was the prognosis of OICD in relation to change in exposure, i.e., change to another job or work tasks. Self-reported changes were reported in ten studies. Among these were one study with focus on the influence of self-reported current exposure and two studies that included the duration of exposure prior to diagnosis.

### Prognostic outcomes

As measurement of prognosis of OCD, the majority of studies used either healing vs persistence and/or various degrees of improvement of skin reactions. One study reported prognostic outcome in severity scores (Jungbauer et al. 2004b).

Prognostic outcome was based on clinical examinations in two studies (Adisesh et al. 2002; Lindemayr 1984), while in one study, clinical assesment of healing was performed on a subgroup of patients (Chia 1991). In the remaining studies, outcome was self-reported.

### Quality of the studies

Low rate of participation at follow-up in four of the studies or missing information on outcome in one study (Lindemayr 1984) may pose the risk of selection bias.

Information on job change, or change of work tasks and the prognostic outcome, was self-reported in most of studies and may present a risk of misclassification of exposure and outcome, probably non-differential. This could lead to an underestimation of beneficial effects of work change if workers who had to change jobs are more likely to report a worse prognosis.

Overall, three studies were graded as high quality (Caroe et al. 2018b; Cvetkovski et al. 2006; Malkonen et al. 2010), four of medium quality, and the remaining of low quality (Table S3).

### Results

The overall proportion of healed ICD varied from 18 to 72%, and the proportion of improved ICD varied from 41 to 84%.

Five studies reported a more favorable prognosis for healing of OCD including ICD for workers who changed occupation (Caroe et al. 2018a, 2018b; Malkonen et al. 2010; Rosen and Freeman 1993) and/or work task (Malkonen et al. 2009; Rosen and Freeman 1993 Australia). Another four studies reported no association between healing and change of occupation (Chia 1991; Keczkes et al. 1983; Lindemayr 1984; Shah et al. 1996).

Malkonen et al. (2010) in a Finnish study that included 251 patients with ICD with a mean follow-up time of 10.5 years reported healing in ICD of 35% with no difference with regard to ACD. Lack of healing was associated with no change of occupation, OR 1.55 (1.03–2.34) and with a dose–response relation to duration of OCD prior to diagnosis. In a large Danish study of workers with OCD with a sub-cohort of 954 workers with ICD due to wet work, Caroe et al. (2018b), (2018c) reported healing and improvement of OCD associated with change of profession or non-employment 4–5 years after diagnosis, with no difference between ICD and ACD. The overall healing of ICD or improvement of ICD was 15% and 52%, respective, among those who stayed in the same profession, 19% and 67% for those who changed profession, and 28% and 61% for those who were outside the labor marked at follow-up. Compared to workers who stayed in their professions, the OR for improvement of ICD was 2.13 (1.49–3.05) for workers who left their professions and 1.79 (1.19–2.70) for those without a job. They found inverse dose–response relations at follow-up between hours spent with wet work as well as frequency of handwashing and healing/improvement of ICD. Each step down in categories of exposure increased the chance of healing by 25% and 34% and of improvement by 4% and 8%.

Another Danish study based on recognized OHE cases, where almost 50% had left their job, demonstrated an overall 1 year improvement rate of 41% and a strong association between baseline severity of OCD and job loss, but no association to job change or duration of OCD (Cvetkovski et al. 2006).

Rosen and Freeman (1993) reported an overall prevalence of healing for 34% and improvement among 70% in an Australian study of 334 patients 1–5 years after diagnoses of OCD, with no difference between ICD and ACD. Healing or improvement was reported in 43% and 76% among patients who changed industry, compared to 28% and 67% for workers staying in the same industry—the RR* of healing was 1.6 (1.2–2.1). This study also reported a more favorable prognosis of workers who stayed in the industry and changed work task (Table 4).

In two studies reporting on the effect of exposure duration prior to diagnosis. Adisesh et al. (2002) found non-improvement of the eczema associated with a higher exposure duration among non-atopics, while Chia (1991) found no association between duration of exposure and healing of ICD.

### Conclusion

Results should be interpreted with caution as most of the included studies were performed on selected populations of patients. Therefore, they probably represent only the most severe cases, and may not be representative for the prognosis of less severe ICD in individuals not seeking specialist medical attention.

With these reservations, the available evidence from epidemiological studies supports a poor prognosis for improvement with complete healing of OCD including ICD if no or non-specific preventive measures are undertaken—the level of evidence was assessed to be strong (+++). The literature supports a better prognosis of complete healing of ICD when reduced exposure is achived by change of occupation or work task, with the level of evidence being moderate (++).

Although a greater proportion of individuals will naturally experience improvement rather than complete healing, quality studies focusing on improvement were sparse and with conflicting information, and the level of evidence found to be limited (+).

The level of evidence for an association between long duration of exposure prior to diagnosis and subsequent continuous ICD was limited because of to few studies and conflicting results (+).

## Discussion

### Summary of main results

We identified and reported results and made quality assessment of 55 epidemiological papers from 48 studies presenting occupational risk factors for ICD and the prognosis of ICD and in addition included supplementary documentation from experimental studies.

Concerning wet work exposure, the available evidence supports an association between wet work and minor ICD in combination with other irritants. No threshold limit could be described. The level of evidence was considered strong (+++).

When, however, exposure was focused on disinfectants and detergents, often in combination with wet work, the overall evidence for a causal association with ICD was considered moderate (++), while the overall evidence for detergent and non-alcoholic disinfectants in combination with general wet work was assessed to be strong (+++).

The overall evidence for a causal association between occlusive glove exposure without other irritants, and ICD, was considered limited (+), while the evidence for a causal association related to a combination of occlusive gloves and other irritant exposure was considered moderate (++).

The evidence for a causal association between exposures to MWF and ICD was considered moderate (++), and the overall evidence for a causal association between mechanical exposures and ICD was considered limited (+).

Regarding the outcome of efforts to heal OICD, the included epidemiological studies support a poor prognosis for complete healing after no or non-specific preventive measures in the work environment, with the level of evidence being strong (+++). A better prognosis for complete healing was found when exposure was ceased due to change of work task—the level of evidence was moderate (++).

While more individuals in the clinical setting experience partial remission rather than complete healing, the studies investigating improvement of OICD in relation to change of occupation or work tasks are inconclusive and evidence is considered limited (+).

### Misclassification of exposure

Since the risk of OICD is expected to be dose-dependent, quantitative onsite exposure information is preferred to qualitative information, reducing the likelyhood of reporting bias which would eventually lead to risk estimates biased toward no effect due to dilution of exposure.

Most of the studies, however, relied on self-reported exposures. Only three studies provided independent quantitative measurements or observations (Daftarian et al. 2002; Kiec-Swierczynska and Wojtczak 2000; Lan et al. 2011), and in three other studies, semi-quantitative exposure assessment was performed by experts or based on self-reported exposures reported in diary cards (Berndt et al. 2000; Jee et al. 1986; Visser et al. 2014b).

Some of the studies relied solely on comparisons between different work groups, e.g., hospital departments, without providing further information regarding the extend of the on exposure—they can primarily be used for hypothesis generation.

A study by Jungbauer et al. (2004a) compared questionnaire and observation-based information regarding wet work, and found that the duration of exposure was overestimated by a factor of approximately two, while the frequency of exposure to wet work was underestimated by about the same factor. The same trend was found by Lund et al. (2019) in professions with a high frequency of wet work; they reported a low validity of self-reported wet work exposures, with sensitivity of 50% and specificity of 60%.

The implication of theese type of non-differential misclassification will probably result in attenuation of the risk of exposure when reported as dichomeous exposure, i.e., wet work more than 2 h/day, while a more detailed analysis showing a dose–response relation would underestimate the risk at lower exposures, because the reported risk would start at a higher exposure than revealed by the actual measurements.

Likewise as self-reported frequencies of exposures are probably underreported, the actual risk would be from higher actual frequencies than reported in the studies.

Two other studies compared observations and self-assessment of exposures to water, gloves, hand disinfectants, and moisturizers among nurses and a mixed group of mechanics, kitchen, and office workers, and reported a tendency to overestimate all exposures (Anveden et al. 2006; Anveden and Meding 2007).

### Misclassification of outcome

A non-differential misclassification of outcome case definitions for ICD with low specificity and overreporting of disease could be expected to dilute any real associations with exposure toward the null, while studies with low sensitivity and underestimation of the disease could be expected not to affect relative risk of disease in relation to exposure, but reduces risk differences (Rothman et al. 2008). Self-reported questionnaire-based HE or symptoms of HE have been validated against clinical examinations in several studies, revealing that self-reported HE tended to underestimate the prevalence of clinical HE in studies with low sensitivity and high specificity, whereas the prevalence of HE based on self-reported symptoms tended to overestimate the prevalence of HE in studies with high sensitivity and low specificity (Carstensen et al. 2006; Livesley et al. 2002; Meding and Barregard 2001; Smit et al. 1992). Bregnhoj et al. (2011b) have recently validated self-reported HE by the Nordic Occupational Skin Questionnaire NOSQ-2002 used by several of the studies reported in this review. In a study, among Danish hairdressing apprentices comparing self-reported against clinical examination, they reported a good agreement, with a sensitivity of 70.3%, specificity of 99.8% and positive/negative predictive values of 96.3/98.5%.

No gold standard for a definition of ICD or irritant HE exists, and in most studies, case definitions of ICD have typically been made clinically as an exclusion diagnosis based on no finding of ACD and an assumed temporal relation to a history of a supposed relevant irritant exposure (Ale and Maibach 2014; Friis et al. 2014). In recent years, however, as discussed by Friis et al. (2014); Schwensen et al. (2014), diagnostic criteria for the diagnosis of ICD and combined ICD and ACD have changed and are now defined by significant exposures to known irritants and the temporal relationship between exposure and the dermatitis, the German wet work criteria as described in the background section. Theese authors also discuss the fundamental problem that the diagnostic criteria for ICD are based on known risk factors and not a valid test, resulting in a mixing of exposures and thus a risk of overestimation of the occurrence of ICD in occupational settings with high exposures to irritants.

Another drawback when exposure is included in the diagnostic criteria is difficulties in determination of exposure response relations in different studies that use different definitions of ICD. This is the case when some studies report on major dermatitis resembling eczema and other minor changes/irritant reactions.

Furthermore, ICD is suspected to play a role in the development of ACD. As dysfunction of the skin barrier is a main feature of any CD, it is reasonable to assume that this disruption may result in increased secondary sensitization rates to allergens, facilitating secondary ACD (Lee et al. 2013).

### Supplementary experimental evidence

In addition to epidemiological studies, experimental studies have been demonstrated to be relevant. Assessment of the irritant potential of chemicals has been investigated trough different methods including visual scoring, transepidermal water loss, laser-Doppler flowmetry and skin color reflectance (Kartono and Maibach 2006). Slotosch et al. (2007) studied the effects of propanol-based disinfectants and detergents on skin irritation, and reported more irritation and barrier disruption for sodium lauryl sulfate (SLS) than for the alcohol-based hand rub and a protective effect of combined use of SLS and disinfectants. A study by Pedersen et al. (2005) found an increased irritant response for detergent as compared to disinfectants alone and disinfectants combined with detergents after daily repeated application of detergents, alcohol-based disinfectants, and detergents/disinfectants.

Regarding gloves, supplementary evidence of experimental studise was gathered in a review by Tiedemann et al. (2015). They concluded that the negative effect of occlusion in itself is limited and that only extensive and long-term occlusion will cause barrier impairment.

For mehcanical exposure to fibers evidence in an experimental setting has been provided by Tsunoda et al. (2014). In a study on volunteers that underwent 24 h of provocation with different continuous glass filament, only transient skin reactions were observed, while no changes were visible after 24 h.

### Atopy and other pre-existing non-occupational factors

Atopic disposition is a well-known vulnerability factor for susceptibility to ICD, with increased OR of around a factor of 3, when the severity corresponds to medically treated atopic disease. Consequently, accounting for atopy, at best in adjusted analyses, has been included in our quality assessment of the studies. However, when it comes to an exposure–response outcome, there was not a substantial difference between studies including information about atopy, and those that did not. In fact, some studies after adjustments for atopy showed stronger associations between exposure to irritants and ICD (Callahan et al. 2013; Visser et al. 2014a). In addition, some studies have indicated a healthy worker effect, fewer susceptible workers entering the work areas with irritant exposures (Bandier et al. 2013; Bregnhoj et al. 2011a). This is in accordance with the common clinical advice given to atopic individuals not to enter risk-trades.

Gender and age were included in most of the studies, but while some studies reported association to female gender and young age, inclusion of those parameters in the analyses in general, did not provide substantial evidence of changes in the effect of occupational irritant exposures. Therefore, an inverse relationship to age, as demonstrated in some studies, could not be confirmed on this review.

Private exposures (housework and domestic childcare) could be relevant additional exposures, which might contribute to the overall exposure burden of irritants along with occupational exposures. However, only a few studies included domestic exposures. While some reported childcare or housework to be significant risk factors for ICD (Bauer et al. 2001; Ibler et al. 2012; Mortz et al. 2014), others fund no association (Held et al. 2001; Lan et al. 2011; Lee et al. 2013) and the available evidence does not allow for estimation of the effect of such exposures (Table S-3 in the online supplementary list details on original studies with focus on individual risk factors).

### Strength and limitations

Although the literature search was broad and performed in several databases, we may have missed studies of relevance not published in peer-reviewed journals or not indexed to accommodate our search strategy. The literature search covers a 40-year period of time and was updated until March 2020.

We were not able to perform any meta-analyses due to differences in the reported outcomes.

To obtain enough information on associations between ICD and irritants, we included studies with very different designs, measures of exposures, diagnostic criteria, and sources of the diagnosis of ICD. We found that many studies were influenced by information bias, resulting in misclassifications of exposure and diagnosis. In most cases, these misclassifications were, however, non-differential causing attenuation of exposure disease associations with smaller effects than would be expected without misclassification. However, differential misclassification might also be present due to the incorporation of exposure in the diagnosis of ICD and this could tend to overestimate risk of ICD in groups with expected higher exposures to irritants, e.g.. wet work, also in studies where ICD has been diagnosed clinically.

The direction of bias due to misclassification could likely cause lower disease association than would be the case without incorrect classifications.

Private exposure from household or leisure time activities is included in only a few studies; this unadjusted additional exposure would tend to overestimate the effect from occupational exposure.

## Conclusion

This review provides strong evidence for associations between irritant exposures and the development of OICD in relation to wet work exposure, with or without combined exposure to detergent and disinfectants. The evidence for metalwork exposures was moderate and limited for exposure to mechanical exposures and gloves. Furthermore, this review provides strong evidence for a poor prognosis of complete healing when exposure continues unchanged, and moderate evidence for a complete healing with cessation or decrease of exposure. Only a few studies investigated improvement rather than complete healing and the results were variable, and therefore, the evidence of partial improvement of ICD in relation to occupational changes was limited.

However, there were few high-quality studies and a number of limitations affected all the included studies in varying degree, making comparison and summation of evidence difficult. These limitations included low diagnostic specificity, non-quantitative exposure information, lack of exposure response data, and to some extent limited confounder adjustment, with atopy presumeably being the most important potential confounder.

The bulk of studies had a cross-sectional design, and there is a need of follow-up studies focusing on ICD with concomitant quantitative exposure assessment and assessment of ICD using well-defined clinical measures.

Wet work is the most prevalent exposure, giving rise to the highest occurrences of absolute numbers of OICD, and occupations with frequent wet work exposure, e.g., in the health and social care sectors, will be increasing in the coming years. Danish hospital nurses typically performed handwashing procedures some 30–40 times per day, but at many hospitals, hand wash with soap is now reduced to less than 4–5 times per day, while the hand hygeine is based on alcoholic disinfectants only. Regarding glove use, we are not able to give specific advise with regard to either the preventive potential or the risk factor in itself. Robust knowledge of prognostic factors is important for clinical practice and should be subject to further studies.

## Supplementary Information

Below is the link to the electronic supplementary material.

Supplementary file1 (DOCX 44 KB)

Supplementary file2 (DOCX 39 KB)

Supplementary file3 (DOCX 106 KB)

Supplementary file4 (DOCX 29 KB)

## Declarations

### Data sharing statement

Request for access to data should be addressed to the corresponding author: gitte.hojbjerg.jacobsen@rsyd.dk.
