# Supplementary material for: Causes of irritant contact dermatitis after occupational skin exposure: a systematic review
Source: Int Arch Occup Environ Health. 2021 Oct 19;95(1):35–65. doi: 10.1007/s00420-021-01781-0 (PMC8755674; doi:10.1007/s00420-021-01781-0)
Supplement: Supplementary file 1 — Supplementary file1 (DOCX 44 KB) [file 420_2021_1781_MOESM1_ESM.docx]

**Table S-1 Documentation of search strategy**

PubMed, search strategy, table and search string. Date of search October 29^th^, 2015

| **Outcome** |  | **Irritative skin exposure** |  | **Work-relation** | **References** |
| --- | --- | --- | --- | --- | --- |
| **#1**  contact dermatitis[MH] OR hand dermatoses[MH] OR eczema*[TW] OR dermatitis[TW] | **AND** | **#4**  irritants[MH] OR irritative[TW] OR irritant*[TW] OR phototoxic*[TW] OR wet work[TW]OR detergents[MH] OR detergent*[TW] OR cutting fluid*[TW] OR "Industrial Oils/adverse effects"[Mesh] | **AND** | **#5**  occupational exposure[MH OR occupational diseases[MH] OR occupational group[MH] OR occupation*[TW] OR industry[MH] OR industry[TW] | **#9**  **1623** |
| **#2**  occupational dermatitis[MH] OR occupational dermatitis[TW] | **AND** | **#4**  irritants[MH] OR irritative[TW] OR irritant*[TW] OR phototoxic*[TW] OR wet work[TW]OR detergents[MH] OR detergent*[TW] OR cutting fluid*[TW] OR "Industrial Oils/adverse effects"[Mesh] |  |  | **#14**  **1114** |
| **#3**  irritant dermatitis[MH] OR Phototoxic dermatitis [MH] OR irritant dermatitis[TW] OR irritant contact dermatitis[TW] |  |  | **AND** | **#5**  occupational exposure[MH OR occupational diseases[MH] OR occupational group[MH] OR occupation*[TW] OR industry[MH] OR industry[TW] | **#11**  **673** |
| **#6**  english[LA] OR german[LA] OR danish |  |  |  | **(#9 OR #10 OR #11) AND #6** | **#12**  **1465** |
|  |  |  |  |  |  |
| **#7**  c*as*e reports[PT] |  |  |  | **#12 NOT #7** | **#13**  **1203** |
| **#8**  Review[PT] |  |  |  | **#13 NOT #8** (*original articles without reviews, no date limits*) | **#14**  **959** |
|  |  |  |  | **#13 AND #8** (*reviews without cases, no date limits*) | **#15**  **244** |
|  |  |  |  | **#12 AND #7 AND #8** (*reviews with cases, no date limits*) | **#16**  **12** |

*#1 contact dermatitis[MH] OR hand dermatoses[MH] OR eczema*[TW] OR dermatitis [TW]*

***#2*** *occupational dermatitis[MH] OR occupational dermatitis[TW]*

*#3 irritant dermatitis[MH] OR Phototoxic dermatitis [MH] OR irritant dermatitis[TW] OR irritant contact dermatitis[TW]*

***#4*** *irritants[MH] OR irritative[TW] OR irritant*[TW] OR phototoxic*[TW] OR wet work[TW]OR detergents[MH] OR detergent*[TW] OR cutting fluid*[TW] OR "Industrial Oils/adverse effects"[Mesh]*

***#5*** *occupational exposure[MH OR occupational diseases[MH] OR occupational group[MH] OR occupation*[TW] OR industry[MH] OR industry[TW]*

***#6*** *english[LA] OR german[LA] OR danish[LA]*

***#7*** *case reports[PT]*

***#8*** *Review[PT]*

***#****9 #1 AND #4 AND #5*

*#10 #2 AND #4*

*#11 #3 AND #5*

*#12 (#9 OR #10 OR #11) AND #6*

*#13 #12 NOT #7*

*#14 #13 NOT #8*

*#15 #13 AND #8*

*#16 #12 AND #7 AND #8*

Embase, search strategy, table and search string. Date of search October 29^th^, 2015

| **Outcome** |  | **Irritative skin exposure** |  | **Work-relation** | **References** |
| --- | --- | --- | --- | --- | --- |
| 1 exp * hand disease/  2 exp *contact dermatitis/  3 (hand adj3 dermatoses) tw  4 (hand adj3 dermatosis).tw.  5 exp ‘eczema/  6 exp *dermatitis  **7: 1 or 2 or 3 or 4 or 5 or 6** | **AND** | 15 irritant agent/  16 irritant*.tw.  17 irritative.tw.  18 Phototoxic.tw.  19 (wet adj3 work).tw.  20 Dertergent/  21 Oil/ae[Adverse Drug Reaction]  22 “industrial oil*2.tw  23 detergent.tw.  24 “cutting fluid*2”.tw.  **25 15 or 16 or 17 or 18 or 19 or 20 or 21 or 22 or 23 or 24** | **AND** | 26 *occupational exposure/  27 exp *occupational disease/  28 exp *named groups by occupation/  29 exp *industry/  30 industr*.tw.  31 occupation*.tw.  **32 26 or 27 or 28 or 29 or 30 or 31** | **(33)**  **1012** |
| 8 occupational eczema/  9 (occupational adj3 dermatitis).tw.  **10**: **8 or 9** | **AND** | 15 irritant agent/  16 irritant*.tw.  17 irritative.tw.  18 Phototoxic.tw.  19 (wet adj3 work).tw.  20 Dertergent/  21 Oil/ae[Adverse Drug Reaction]  22 “industrial oil*2.tw  23 detergent.tw.  24 “cutting fluid*2”.tw.  **25: 15 or 16 or 17 or 18 or 19 or 20 21 or 22 or 23 or 24** |  |  | **(34)**  **839** |
| 11 irritant dermatitis/  12 (irritatant adj3 dermatitis).tw.  13 (phototoxic adj3 dermatitis).tw.  **14: 11 or 12 or 13** |  |  | **AND** | 26 *occupational exposure/  27 exp *occupational disease/  28 exp *named groups by occupation/  29 exp *industry/  30 industr*.tw.  31 occupation*.tw.  **32: 26 or 27 or 28 or 29 or 30 or 31** | **(35)**  **819** |
|  |  |  |  | **33 or 34 or 35** | **(36)**  **1626** |
|  |  |  |  | Limits Danish or English or German | **(37)**  **1371** |
|  |  |  |  |  |  |
| 38 case report/ |  |  |  | **37 not 38** | **(39)**  **1232** |
| 40 review/ |  |  |  | **39 not 40** (*original articles without reviews, no date limits*) | **(41)**  **1001** |
|  |  |  |  | **39 and 40** (*reviews without cases, no date limits*) | **(42)**  **231** |
|  |  |  |  | **37 and 38 and 40** (*reviews with cases, no date limits*) | **(43)**  **6** |

1. exp * hand disease/
2. exp *contact dermatitis/
3. (hand adj3 dermatoses) tw
4. (hand adj3 dermatosis).tw.
5. exp ‘eczema/
6. exp *dermatitis
7. 1 or 2 or 3 or 4 or 5 or 6
8. occupational eczema/
9. (occupational adj3 dermatitis).tw.
10. 8 or 9
11. irritant dermatitis/
12. (irritatant adj3 dermatitis).tw.
13. 13 (phototoxic adj3 dermatitis).tw.
14. 11 or 12 or 13
15. irritant agent/
16. irritant*.tw.
17. irritative.tw.
18. Phototoxic.tw.
19. (wet adj3 work).tw
20. Dertergent/
21. Oil/ae[Adverse Drug Reaction]
22. “industrial oil*2.tw
23. detergent.tw.
24. 24 “cutting fluid*2”.tw.
25. 15 or 16 or 17 or 18 or 19 or 20 or 21 or 22 or 23 or 24
26. *occupational exposure/
27. exp *occupational disease/
28. exp *named groups by
29. exp *industry/
30. industr*.tw.
31. occupation*.tw.
32. 26 or 27 or 28 or 29 or 30 or 31
33. 7 and 25 and 32
34. 10 and 25
35. 14 and 32
36. 33 or 34 or 35
37. Limit 36 to (danish or English or german)
38. case report/
39. 37 not 38
40. review/
41. 39 not 40
42. 39 and 40
43. 37 and 38 and 40

Web of Science, search strategy, table, search string. Date of search November 5’th 2015.

| **Outcome** |  | **Irritative skin exposure** |  | **Work-relation** | **References** |
| --- | --- | --- | --- | --- | --- |
| #1 TS=(contact NEAR/3 dermatitis)  #2 TS=(hand NEAR/3 dermatoses)  #3 TS=(hand NEAR/3 dermatosis)  #4 Topic: (eczema*) OR topic: (dermatitis)  **#5**= **#1 or #2 or #3 or #4** | **AND** | #8 TS= (irritant*) OR TS= (irritative) OR TS= (phototoxic*) OR TS=(detergent*)  #9 TS=(wet NEAR/3 work) OR TS=(cutting NEAR/3 fluid*) OR TS=(industrial NEAR/3 oil SAME adverse)  **#10= #8 or #9** | **AND** | **#11** TS=(occupational NEAR/3 exposure) OR TS=(occupational NEAR/disease*) OR TS=( occupation*) OR TS=(industry) | **#12**  **815** |
| **#6** TS=(occupational NEAR/3 dermatitis) | **AND** | #8 topic: (irritant*) OR topic: (irritative) OR topic: (phototoxic*) OR topic (detergent*)  #9 TS=(wet NEAR/3 work) OR TS=(cutting NEAR/3 fluid*) OR TS=(industrial NEAR/3 oil SAME adverse)  **#10**= **#8 or #9** |  |  | **#13**  **402** |
| **#7** TS=(irritant NEAR/3 dermatits) OR TS=((phototoxic NEAR/3 dermatitis) OR TS=(irritant contact dermatitis) |  |  | **AND** | **#11** TS=(occupational NEAR/3 exposure) OR TS=(occupational NEAR/disease*) OR TS=( occupation*) OR TS=(industry) | **#14**  **521** |
|  |  |  |  | **#12 OR #13 OR #14** | **#15**  **815** |
|  |  |  |  | **#15 AND LA=(English OR German)** | **#16**  **763** |
|  |  |  |  | **#16 NOT DT=(review)** | **#17**  **666** |
|  |  |  |  | **#16 AND DT=(review)** | **#18**  **97** |
|  |  |  |  | **#16 NOT DT=(review) Refined by:** [excluding] **DOCUMENT TYPES:**( PROCEEDINGS PAPER OR MEETING ABSTRACT OR HARDWARE REVIEW OR NOTE OR EDITORIAL MATERIAL OR LETTER) | **#19**  **587** |

OSH-UPDATE, Databases HSELINE, NIOSHTIC, CISDOC and RILOSH. Search strategy, table, search string. No limits have been added

Date of search November 3’th 2015

| **Outcome** |  | **Irritative skin exposure** |  | **Work-relation** | **References** |
| --- | --- | --- | --- | --- | --- |
| **#1**  GW{contact dermatitis} OR GW{eczema*} OR GW{dermatitis} OR GW{hand dermatoses} |  | **#4**  GW{irritant*} OR GW{irritative} OR GW{phototoxic*} OR GW{wet work} OR GW{detergent*} OR GW{cutting fluid} OR GW{industrial oil**}** |  | **#5**  GW{occupational exposure} OR GW{occupational disease*} OR GW{industry} | **#6:**  **1646** |
| **#2**  GW{occupational dermatitis} | AND | **#4**  GW{irritant*} OR GW{irritative} OR GW{phototoxic*} OR GW{wet work} OR GW{detergent*} OR GW{cutting fluid} OR GW{industrial oil} |  |  | **#7**  **557** |
| **#3**  GW{irritant dermatitis} OR GW{phototoxic dermatitis}OR GW{irritant contact dermatitis} |  |  | AND | **#5**  GW{occupational exposure} OR GW{occupational disease*} OR GW{industry**}** | **#8**  **354** |
|  |  |  |  | **#6 OR #7 OR #8** | **#9**  **1877** |
| #10  DC{OUHSEL} OR DC{OUCISD} OR DC{OUNIOC} OR DC{OURILO} |  |  |  | **#9 AND #10** | **#11**  **1406** |

Search string.

Step: Hits: Strategy:

#1 13807 GW{contact dermatitis} OR GW{eczema*} OR GW{dermatitis} OR GW{hand dermatoses}

#2 1999 GW{occupational dermatitis}

#3 791 GW{irritant dermatitis} OR GW{phototoxic dermatitis} OR GW{irritant contact dermatitis}

#4 17556 GW{irritant*} OR GW{irritative} OR GW{phototoxic*} OR GW{wet work} OR GW{deter gent*} OR GW{cutting fluid} OR GW{industrial oil}

#5 144103 GW{occupational exposure} OR GW{occupational disease*} OR GW{industry}

#6 1646 #1 AND #4 AND #5

#7 557 #2 AND #4

#8 354 #3 AND #5

#9 1877 #6 OR #7 OR #8

#10 685428 DC{OUHSEL} OR DC{OUCISD} OR DC{OUNIOC} OR DC{OURILO}

#11  **1406** #9 AND #10

All databases, original papers

| Pubmed | PubMed 1959-2015 | 959 |
| --- | --- | --- |
|  | ≥ 1980 | 862 |
|  | Dublets | 3 |
|  | Titles database from pubmed | **859** |
| Embase | 1959-2015 | 1001 |
|  | ≥1980 | 915 |
|  | Dublets Embase | 16 |
|  | Dublets PubMed | 512 |
|  | Conference abstracts | 169 |
|  | Titles database from Embase | **218** |
| Web of Science  (WOS) | 1934-2015 | 587 |
|  | ≥1980 | 585 |
|  | Dublets Pubmed and Embase | 428 |
|  | Case reports | 50 |
|  | Titles database from WOS | **107** |
| OSH-  UPDATE | 1922-2011 | 1406 |
|  | ≥1980 | 1113 |
|  | Dublets OSH-UPDATE | 108 |
|  | Dublets other databases | 248 |
|  | Language | 90 |
|  | Case reports | 170 |
|  | Conference abstracts, pamphlets, evaluation reports | 260 |
|  | Reviews | 48 |
|  | Titles database | **189** |
| Selection | Titles all databases | **1373** |
|  | Excluded title | 850 |
|  | Included title | 523 |
|  | No abstract | 37 |
|  | Excluded abstract | 303 |
|  | Included abstract | 183 |
|  | Excluded article | 135 |
|  | Included article | 48 |
|  | “Snow-ball articles” | 2 |
|  | Articles included | **50** |

Repeated PubMed Search 2019 and 2020

| Selection | Titles PubMed | **141** |
| --- | --- | --- |
|  | Excluded titles | 93 |
|  | Included titles | 48 |
|  | No abstract | 1 |
|  | Excluded abstract | 32 |
|  | Included abstract | 15 |
|  | Excluded article | 10 |
|  | Included article | **5** |
