# Supplementary material for: Causes of irritant contact dermatitis after occupational skin exposure: a systematic review
Source: Int Arch Occup Environ Health. 2021 Oct 19;95(1):35–65. doi: 10.1007/s00420-021-01781-0 (PMC8755674; doi:10.1007/s00420-021-01781-0)
Supplement: Supplementary file 2 — Supplementary file2 (DOCX 39 KB) [file 420_2021_1781_MOESM2_ESM.docx]

**Table S2** Data extraction tables

| **Reference** | Ref-id |  |
| --- | --- | --- |
|  | Author |  |
|  | Year |  |
|  | Study – ICD exposure |  |
|  | Study – ICD prognosis |  |
|  | Study– indiv. risk factors |  |
|  | Other, commentary |  |
| **Study design** | □ Cohort- prospective |  |
|  | □ Cohort- retrospective |  |
|  | □ case control |  |
|  | □ cross sectional |  |
|  | other, specify |  |
| **Population**  **(specify for exposed and controls)**  **(participation rate)** | Country |  |
|  | □ General population |  |
|  | □ Industry workers |  |
|  | □ Convenience Sample |  |
|  | Other, specify |  |
| **Men, exposed/**  **controls** | n= |  |
|  | Average age |  |
|  | Range Age |  |
|  | Remarks |  |
| **Woman, exposed /**  **controls** | n= |  |
|  | Average age |  |
|  | Range Age |  |
|  | Remarks |  |
| **Total, exposed/**  **controls** | n= |  |
|  | Average age |  |
|  | Range Age |  |
|  | Remarks |  |
| **Quantitative exposure assessment** | Measured Yes/no |  |
|  | Conc range |  |
|  | Total/average conc |  |
|  | □individual measurements |  |
|  | □ group measurements |  |
|  | □ area measurement |  |
|  | Other specify |  |
| **Semi-Quantitative exposure assessment** | □ expert judgement |  |
|  | □ JEM |  |
| **qualitative exposure assessment** | □ self-report |  |
|  | □ home |  |
|  | □ industry |  |
|  | □ occupation |  |
| **Remarks exposure assessment** |  |  |
| **Type of Exposure** |  |  |
| **More exposures** |  |  |
| **Type of occupation** |  |  |
| **Diagnosis** | Diagnosis /Outcome |  |
|  | □ clinical |  |
|  | □ self-report |  |
|  | □ register based |  |
|  | □ industry |  |
|  | □ occupation |  |
|  | Diagnostic criteria used |  |
|  | Remarks |  |
| **Men** | OR (CI) |  |
|  | RR (CI) |  |
| **Woman** | OR (CI) |  |
|  | RR (CI) |  |
| **Total** | OR (CI) |  |
|  | RR (CI) |  |
| **Dose-response performed?** | yes/no |  |
|  | Range exposure /conc. /time/times |  |
|  | RR (CI) for exp. conc/time etc. |  |
| **Covariates adjusted for** | □ allergy /patch test relevant allergens |  |
|  | □ atopy |  |
|  | □ gender |  |
|  | □ age |  |
|  | □ private exposures |  |
|  | others, specify |  |
| **the exposure adequately described?** | □ yes |  |
|  | □ yes, partly |  |
|  | □ no |  |
|  | □ no mention |  |
|  | □ n/a (ikke relevant) |  |
|  | □ do not know |  |
| **Is the outcome adequately described?** | □ yes |  |
|  | □ yes, partly |  |
|  | □ no |  |
|  | □ no mention |  |
|  | □ n/a (not relevant) |  |
|  | □ do not know |  |
| **Was the measurement of the outcome sound?** | □ yes |  |
|  | □ yes, partly |  |
|  | □ no |  |
|  | □ no mention |  |
|  | □ n/a (not relevant) |  |
|  | □ do not know |  |
| **Adequately corrected for confounders?** | □ yes |  |
|  | □ no |  |
|  | □ n/a (not relevant) |  |
|  | □ do not know |  |
|  | If no data probably confounded by: |  |
|  |  |  |
| **Data probably biased?** | □ no |  |
|  | □ yes, partly |  |
|  | □ yes |  |
|  | □ misclassification  of exposure |  |
|  | □ misclassification  of outcome |  |
|  | □selection of  study population |  |
|  | □other, specify: |  |
| **Are the statistical analyses appropriate?** | □ yes |  |
|  | □ yes, partly |  |
|  | □ no |  |
|  | □do not know |  |
| **Are the results probably due to chance?** | □ yes |  |
|  | □ yes, partly (CI contains 1 or p-value >0,05) |  |
|  | □ no |  |
| **Grading of the study** | 1 (best) |  |
|  | 2 |  |
|  | 3 |  |
|  | 4 |  |
|  | 5 (not suitable) |  |

| Quality Scores | Score 1 | Score 0 |  |
| --- | --- | --- | --- |
| Study | Cohort study or case control study with  population or hospital controls | Case control study with convenience controls. Cross- sectional studies |  |
| Number of participants | >=75 cases | <75 cases |  |
| Response rate | > 60% | ≤ 60% |  |
| source of exposure information | non-self-reports | self- reports |  |
| exposure measure: | quantitative or semi-quantitative | qualitative |  |
| source of diagnosis | Hospital/clinical | vs. surveillance schemes, self-reported or not well-defined sources |  |
| diagnosis | well defined diagnostic criteria | other criteria |  |
| possible  confounding | accounted for atopy, age and sex in adjusted analyses or by matching | no account atopy, age and sex. |  |
