# Supplementary material for: Causes of irritant contact dermatitis after occupational skin exposure: a systematic review
Source: Int Arch Occup Environ Health. 2021 Oct 19;95(1):35–65. doi: 10.1007/s00420-021-01781-0 (PMC8755674; doi:10.1007/s00420-021-01781-0)
Supplement: Supplementary file 3 — Supplementary file3 (DOCX 106 KB) [file 420_2021_1781_MOESM3_ESM.docx]

**Table S3 Quality assessment of 48 studies, 55 papers presenting analyses of association of ICD or prognosis in relation to work exposures. Grading criteria described below. The final grading is a summary of the epidemiological assessment.**

| **Author** | | **Study design** | **Dimen-sion** | **Respon-se rate** | **Exposure source** | **Exposure measure** | **Outcome source** | **Outcome measure** | **Confoun-der control** | **Total Score** | **Grading of study** | **Work Exposure** |
| --- | --- | --- | --- | --- | --- | --- | --- | --- | --- | --- | --- | --- |
| **Table 1** | |  |  |  |  |  |  |  |  |  |  |  |
| de Boer et al. (1989) | | 0 | 1 | n.r | 0 | 0 | 1 | 1 | 1 | 4 | 3 | Metal work |
| Fischer and Rystedt (1985) | | 0 | 1 | 1 | 1 | 0 | 0 | 1 | 0 | 4 | 4 | Metal work |
| Guo et al. (1994) | | 0 | 0 | 1 | 0 | 0 | 1 | 1 | 0 | 3 | 3 | Hairdressers |
| Held et al. (2001) | | 1 | 0 | 1 | 0 | 0 | 1 | 1 | 1 | 5 | 3 | HCW |
| Jee et al. (1986) | | 0 | 0 | n.r | 1 | 1 | 1 | 1 | 0 | 4 | 3 | Metal work |
| Kavli and Moseng (1987) | | 0 | 0 | 1 | 1 | 0 | 1 | 1 | 0 | 4 | 4 | Food industry |
| Kiec-Swierczynska and Wojtczak (2000) | | 0 | 1 | n.r | 1 | 1 | 1 | 1 | 0 | 5 | 2 | Mineral fibres |
| Stingeni et al. (1995) | | 0 | 1 | 1 | 0 | 0 | 1 | 1 | 0 | 4 | 4 | HCW |
| Stingeni et al. (1996) | | 0 | 1 | 1 | 0 | 0 | 1 | 1 | 0 | 4 | 4 | Gloves |
| Tacke et al. (1995) | | 1 | 1 | n.r | 1 | 0 | 0 | 1 | 0 | 4 | 3 | Food industry |
| Teo et al. (2009) | | 0 | 0 | 1 | 0 | 0 | 1 | 1 | 1 | 4 | 3 | Food industry |
| **Table 2** | |  |  |  |  |  |  |  |  |  |  |  |
| Apfelbacher et al. (2010) | | 1 | 0 | 1 | 0 | 0 | 0 | 0 | 1 | 3 | 3 | Metal work |
| Bauer et al. (2001); (1998) | | 1 | 0 | 1 | 0 | 0 | 1 | 0 | 1 | 4 | 3 | Food industry |
| Berndt et al. (2000) | | 1 | 0 | 1 | 1 | 1 | 1 | 0 | 1 | 6 | 2 | Metal work |
| Callahan et al. (2013) | | 1 | 0 | 1 | 0 | 0 | 1 | 0 | 1 | 4 | 2 | HCW |
| Chou et al. (2004) | | 0 | 0 | n.r | 1 | 0 | 1 | 0 | 0 | 2 | 3 | CS_2_, H_2_SO_4_ |
| Goh and Gan (1994) | | 0 | 0 | n.r. | 1 | 0 | 1 | 0 | 1 | 3 | 4 | Metal work |
| Uter et al. (1998a); (1998b; 1999a; 1999b) | | 1 | 1 | 1 | 0 | 0 | 1 | 0 | 1 | 5 | 2 | Hairdressers |
| Vermeulen et al. (2001) | | 0 | 0 | 1 | 0 | 0 | 1 | 0 | 1 | 3 | 3 | Wet work |
|  |  |  |  |  | 1 |  |  |  |  | 4 |  | Gloves |
| Weistenhofer et al. (2015) | | 0 | 1 | 1 | 1 | 0 | 1 | 0 | 1 | 5 | 2 | Gloves |
| Weistenhofer et al. (2017) | | 0 | 1 | 1 | 0 | 0 | 0 | 1 | 1 | 4 | 2 | Gloves |
| **Table 3** | |  |  |  |  |  |  |  |  |  |  |  |
| Avnstorp (1991) | | 1 | 0 | 0 | 0 | 0 | 0 | 0 | 0 | 1 | 4 | Cement |
| Daftarian et al. (2002) | | 0 | 0 | 0 | 1 | 1 | 0 | 0 | 0 | 2 | 4 | TDI |
| Douwes et al. (2017) | | 0 | 1 | 1 | 0 | 1 | 0 | 0 | 1 | 4 | 3 | Cleaners |
| Flyvholm et al. (2007) | | 0 | 1 | 1 | 0 | 0 | 0 | 0 | 0 | 2 | 4 | HCW |
| Hamnerius et al. (2018) | | 0 | 1 | 1 | 0 | 1 | 0 | 0 | 1 | 4 | 3 | HCW |
| Ibler et al. (2012) | | 0 | 1 | 1 | 0 | 0 | 0 | 0 | 0 | 2 | 4 | HCW/ |
| Jung et al. (2014) | | 0 | 1 | 1 | 0 | 0 | 0 | 0 | 0 | 2 | 4 | Hairdressers |
| Lan et al. (2011) | Study 1 | 0 | 1 | 1 | 0 | 1 | 0 | 0 | 1 | 4 | 3 | HCW |
|  | Study 2 | 0 | 0 | 1 | 1 | 1 | 0 | 0 | 1 | 4 | 2 | HCW |
| Lazarov et al. (2005) | | 0 | 1 | 1 | 0 | 0 | 0 | 0 | 0 | 2 | 4 | Wet work |
| Lee et al. (2013) | | 0 | 1 | 1 | 0 | 0 | 0 | 0 | 1 | 3 | 3 | HCW |
| Mirabelli et al. (2009) | | 1 | 0 | n.r. | 0 | 0 | 0 | 0 | 0 | 1 | 3 | MWF |
| Mirabelli et al. (2012) | | 0 | 1 | 0 | 0 | 0 | 0 | 0 | 0 | 1 | 4 | Cleaners |
| Mortz et al. (2014) | | 1 | 1 | 1 | 0 | 0 | 0 | 0 | 1 | 4 | 3 | Wet work |
| Nielsen (1996) | | 0 | 1 | 0 | 0 | 0 | 0 | 0 | 0 | 1 | 4 | Cleaners |
| Visser et al. (2014b) | | 1 | 1 | 1 | 0 | 1 | 0 | 0 | 0 | 4 | 3 | HCW |
| Visser et al. (2014a) | | 1 | 1 | 1 | 0 | 1 | 0 | 0 | 1 | 5 | 3 | HCW |
| Petersen and Sabroe (1991) | | 0 | 1 | 1 | 0 | 0 | 0 | 0 | 0 | 2 | 3 | Mineral fibres |
| **Table 4 Prognosis** | |  |  |  |  |  |  |  |  |  |  |  |
| Adisesh et al. (2002) | | 1 | 1 | 1 | 0 | n.a | 1 | 0 | 1 | 5 | 3 | Various |
| Caroe et al. (2018a); (2018b) | | 1 | 1 | 0 | 0 | 1 | 0 | 1 | 1 | 5 | 2 | Wet work/various |
| Chia (1991) | | 1 | 1 | 1 | 0 | n.a. | 0 | 1 | 0 | 4 | 3 | Various |
| Cvetkovski et al. (2006) | | 1 | 1 | 1 | 0 | n.a. | 0 | 1 | 1 | 5 | 2 | Various |
| Jungbauer et al. (2004) | | 1 | 1 | 1 | 0 | n.a | 0 | 1 | 0 | 4 | 4 | Various |
| Keczkes et al. (1983) | | 1 | 1 | 0 | 0 | n.a. | 0 | 1 | 1 | 3 | 4 | Various |
| Lindemayr (1984) | | 0 | 0 | 1 | 0 | n.a. | 1 | 0 | 0 | 2 | 4 | Hairdressers |
| Malkonen et al. (2010); (2009) | | 1 | 1 | 1 | 0 | n.a | 0 | 1 | 1 | 5 | 2 | Various |
| Rosen and Freeman (1993 Australia) | | 1 | 1 | 1 | 0 | n.a. | 0 | 1 | 1 | 5 | 3 | Various |
| Shah et al. (1996) | | 1 | 0 | 1 | 0 | n.a. | 0 | 1 | 0 | 3 | 4 | Metal-workers |
| Vester et al. (2012) | | 1 | 1 | 1 | 0 | n.a. | 0 | 1 | 0 | 4 | 3 | Various |

Adisesh A, Meyer JD, Cherry NM (2002) Prognosis and work absence due to occupational contact dermatitis. Contact dermatitis 46(5):273-279 doi:cod460505 [pii]

Apfelbacher CJ, Funke U, Radulescu M, Diepgen TL (2010) Determinants of current hand eczema: results from case-control studies nested in the PACO follow-up study (PACO II). Contact dermatitis 62(6):363-370 doi:10.1111/j.1600-0536.2010.01729.x [doi]

Avnstorp C (1991) Risk factors for cement eczema. Contact dermatitis 25(2):81-88

Bauer A, et al. (2001) Occupational hand dermatitis in food industry apprentices: results of a 3-year follow-up cohort study. International archives of occupational and environmental health 74(6):437-442

Bauer A, et al. (1998) Development of occupational skin diseases during vocational training in baker and confectioner apprentices: a follow-up study. Contact dermatitis 39(6):307-311

Berndt U, Hinnen U, Iliev D, Elsner P (2000) Hand eczema in metalworker trainees--an analysis of risk factors. Contact dermatitis 43(6):327-332

Callahan A, et al. (2013) Winter season, frequent hand washing, and irritant patch test reactions to detergents are associated with hand dermatitis in health care workers. Dermatitis : contact, atopic, occupational, drug 24(4):170-175 doi:10.1097/DER.0b013e318290c57f [doi]

Caroe TK, Ebbehoj NE, Bonde JPE, Flachs EM, Agner T (2018a) Hand eczema and wet work: dose-response relationship and effect of leaving the profession. Contact dermatitis 78(5):341-347 doi:10.1111/cod.12934 [doi]

Caroe TK, Ebbehoj NE, Bonde JPE, Vejlstrup SG, Agner T (2018b) Job change facilitates healing in a cohort of patients with occupational hand eczema. The British journal of dermatology 179(1):80-87 doi:10.1111/bjd.16095 [doi]

Chia (1991) Prognosis of Occupational Dermatitis in Singapore Workers. American journal of contact dermatitis 2(2):105; 105-109; 109 doi:pmid:

Chou TC, Sheu HM, Chiu JE, Wu JD, Shih TS, Chang HY (2004) Combined exposure to carbon disulfide and sulfuric acid simultaneously increases the risk of hand dermatitis in rayon industry. Journal of exposure analysis and environmental epidemiology 14(7):551-557 doi:10.1038/sj.jea.7500385 [doi]

Cvetkovski RS, Zachariae R, Jensen H, Olsen J, Johansen JD, Agner T (2006) Prognosis of occupational hand eczema: a follow-up study. Archives of Dermatology 142(3):305-311 doi:142/3/305 [pii]

Daftarian HS, Lushniak BD, Reh CM, Lewis DM (2002) Evaluation of self-reported skin problems among workers exposed to toluene diisocyanate (TDI) at a foam manufacturing facility. Journal of occupational and environmental medicine / American College of Occupational and Environmental Medicine 44(12):1197-1202

de Boer EM, van Ketel WG, Bruynzeel DP (1989) Dermatoses in metal workers. (I). Irritant contact dermatitis. Contact dermatitis 20(3):212-218

Douwes J, et al. (2017) Determinants of hand dermatitis, urticaria and loss of skin barrier function in professional cleaners in New Zealand. International journal of occupational and environmental health 23(2):110-119 doi:10.1080/10773525.2018.1427307 [doi]

Fischer T, Rystedt I (1985) Hand eczema among hard-metal workers. American Journal of Industrial Medicine 8(4-5):381-394

Flyvholm MA, Bach B, Rose M, Jepsen KF (2007) Self-reported hand eczema in a hospital population. Contact dermatitis 57(2):110-115

Goh CL, Gan SL (1994) The incidence of cutting fluid dermatitis among metalworkers in a metal fabrication factory: a prospective study. Contact dermatitis 31(2):111-115

Guo YL, Wang BJ, Lee JY, Chou SY (1994) Occupational hand dermatoses of hairdressers in Tainan City. Occupational and environmental medicine 51(10):689-692

Hamnerius N, Svedman C, Bergendorff O, Bjork J, Bruze M, Ponten A (2018) Wet work exposure and hand eczema among healthcare workers: a cross-sectional study. The British journal of dermatology 178(2):452-461 doi:10.1111/bjd.15813 [doi]

Held E, Wolff C, Gyntelberg F, Agner T (2001) Prevention of work-related skin problems in student auxiliary nurses: an intervention study. Contact dermatitis 44(5):297-303 doi:cod440509 [pii]

Ibler KS, Jemec GB, Agner T (2012) Exposures related to hand eczema: a study of healthcare workers. Contact dermatitis 66(5):247-253 doi:10.1111/j.1600-0536.2011.02027.x [doi]

Jee SH, Wang JD, Sun CC, Chao YF (1986) Prevalence of probable kerosene dermatoses among ball-bearing factory workers. Scandinavian journal of work, environment & health 12(1):61-65 doi:2182 [pii]

Jung PK, et al. (2014) The effect of work characteristics on dermatologic symptoms in hairdressers. Annals of occupational and environmental medicine 26:13-4374-26-13. eCollection 2014 doi:10.1186/2052-4374-26-13 [doi]

Jungbauer FH, van der Vleuten P, Groothoff JW, Coenraads PJ (2004) Irritant hand dermatitis: severity of disease, occupational exposure to skin irritants and preventive measures 5 years after initial diagnosis. Contact dermatitis 50(4):245-251 doi:10.1111/j.0105-1873.2004.00347.x [doi]

Kavli G, Moseng D (1987) Contact urticaria from mustard in fish-stick production. Contact dermatitis 17(3):153-155

Keczkes K, Bhate SM, Wyatt EH (1983) The outcome of primary irritant hand dermatitis. The British journal of dermatology 109(6):665-668

Kiec-Swierczynska M, Wojtczak J (2000) Occupational ceramic fibres dermatitis in Poland. Occupational medicine (Oxford, England) 50(5):337-342

Lan CC, et al. (2011) Hand dermatitis among university hospital nursing staff with or without atopic eczema: assessment of risk factors. Contact dermatitis 64(2):73-79 doi:10.1111/j.1600-0536.2010.01813.x [doi]

Lazarov A, Nevo K, Pardo A, Froom P (2005) Self-reported skin disease in hydrotherapists working in swimming pools. Contact dermatitis 53(6):327-331

Lee SW, Cheong SH, Byun JY, Choi YW, Choi HY (2013) Occupational hand eczema among nursing staffs in Korea: Self-reported hand eczema and contact sensitization of hospital nursing staffs. Journal of Dermatology 40(3):182-187

Lindemayr H (1984) Eczema in hairdressers. Dermatosen in Beruf und UmweltOccupation and environment 32(1):5-13

Malkonen T, et al. (2010) Long-term follow-up study of occupational hand eczema. The British journal of dermatology 163(5):999-1006 doi:10.1111/j.1365-2133.2010.09987.x [doi]

Malkonen T, et al. (2009) A 6-month follow-up study of 1048 patients diagnosed with an occupational skin disease. Contact dermatitis 61(5):261-268 doi:10.1111/j.1600-0536.2009.01611.x [doi]

Mirabelli MC, et al. (2012) Occupational risk factors for hand dermatitis among professional cleaners in Spain. Contact dermatitis 66(4):188-196 doi:10.1111/j.1600-0536.2011.02023.x [doi]

Mirabelli MC, et al. (2009) Metalworking exposures and persistent skin symptoms in the ECRHS II and SAPALDIA 2 cohorts. Contact dermatitis 60(5):256-263 doi:10.1111/j.1600-0536.2009.01525.x [doi]

Mortz CG, Bindslev-Jensen C, Andersen KE (2014) Hand eczema in The Odense Adolescence Cohort Study on Atopic Diseases and Dermatitis (TOACS): prevalence, incidence and risk factors from adolescence to adulthood. The British journal of dermatology 171(2):313-323 doi:10.1111/bjd.12963 [doi]

Nielsen J (1996) The occurrence and course of skin symptoms on the hands among female cleaners. Contact dermatitis 34(4):284-291

Petersen R, Sabroe S (1991) Irritative symptoms and exposure to mineral wool. American Journal of Industrial Medicine 20(1):113-122

Rosen RH, Freeman S (1993) Prognosis of occupational contact dermatitis in New South Wales, Australia. Contact dermatitis 29(2):88-93

Shah M, Lewis FM, Gawkrodger DJ (1996) Prognosis of Occupational Hand Dermatitis in Metalworkers. Contact dermatitis 34(1):27-30

Stingeni L, Lapomarda V, Lisi P (1995) Occupational hand dermatitis in hospital environments. Contact dermatitis 33(3):172-176

Stingeni L, Lapomarda V, Lisi P (1996) Undesirable effects from latex gloves in hospital health-care personnel. Journal of the European Academy of Dermatology and Venereology 7(1):44-48

Tacke J, Schmidt A, Fartasch M, Diepgen TL (1995) Occupational contact dermatitis in bakers, confectioners and cooks. A population-based study. Contact dermatitis 33(2):112-117

Teo S, Teik-Jin Goon A, Siang LH, Lin GS, Koh D (2009) Occupational dermatoses in restaurant, catering and fast-food outlets in Singapore. Occupational medicine (Oxford, England) 59(7):466-471 doi:10.1093/occmed/kqp034 [doi]

Uter W, Pfahlberg A, Gefeller O, Schwanitz HJ (1998a) Prevalence and incidence of hand dermatitis in hairdressing apprentices: results of the POSH study. Prevention of occupational skin disease in hairdressers. International archives of occupational and environmental health 71(7):487-492

Uter W, Pfahlberg A, Gefeller O, Schwanitz HJ (1998b) Risk factors for hand dermatitis in hairdressing apprentices. Results of the 'Prevention of occupational skin disease in hairdressers' study. Dermatosen in Beruf und Umwelt 46(4):151-158

Uter W, Pfahlberg A, Gefeller O, Schwanitz HJ (1999a) Hand dermatitis in a prospectively-followed cohort of hairdressing apprentices: final results of the POSH study. Prevention of occupational skin disease in hairdressers. Contact dermatitis 41(5):280-286

Uter W, Pfahlberg A, Gefeller O, Schwanitz HJ (1999b) Risk of hand dermatitis among hairdressers versus office workers. Scandinavian journal of work, environment & health 25(5):450-456 doi:459 [pii]

Vermeulen R, Kromhout H, Bruynzeel DP, de Boer EM, Brunekreef B (2001) Dermal exposure, handwashing, and hand dermatitis in the rubber manufacturing industry. Epidemiology (Cambridge, Mass) 12(3):350-354

Vester L, Thyssen JP, Menne T, Johansen JD (2012) Consequences of occupational food-related hand dermatoses with a focus on protein contact dermatitis. Contact dermatitis 67(6):328-333

Visser MJ, et al. (2014a) Filaggrin loss-of-function mutations and atopic dermatitis as risk factors for hand eczema in apprentice nurses: part II of a prospective cohort study. Contact dermatitis 70(3):139-150 doi:10.1111/cod.12139 [doi]

Visser MJ, Verberk MM, van Dijk FJ, Bakker JG, Bos JD, Kezic S (2014b) Wet work and hand eczema in apprentice nurses; part I of a prospective cohort study. Contact dermatitis 70(1):44-55 doi:10.1111/cod.12131 [doi]

Weistenhofer W, Uter W, Drexler H (2017) Protection during production: Problems due to prevention? Nail and skin condition after prolonged wearing of occlusive gloves. Journal of toxicology and environmental healthPart A 80(7-8):396-404 doi:10.1080/10937404.2017.1304741 [doi]

Weistenhofer W, Wacker M, Bernet F, Uter W, Drexler H (2015) Occlusive gloves and skin conditions: is there a problem? Results of a cross-sectional study in a semiconductor company. British Journal of Dermatology 172(4):1058-1065
