# Supplementary material for: Causes of irritant contact dermatitis after occupational skin exposure: a systematic review
Source: Int Arch Occup Environ Health. 2021 Oct 19;95(1):35–65. doi: 10.1007/s00420-021-01781-0 (PMC8755674; doi:10.1007/s00420-021-01781-0)
Supplement: Supplementary file 4 — Supplementary file4 (DOCX 29 KB) [file 420_2021_1781_MOESM4_ESM.docx]

### S4 Evidence criteria causal association

Degree of evidence of a causal association between an exposure to a specific risk factor and a specific outcome. Criteria of the Scientific Committee of the Danish Society of Occupational and Environmental Medicine.

**Description of categories:**

Strong evidence of a causal association (+++):
A causal relationship is very likely. A positive relationship between exposure to the risk factor and the outcome has been observed in several epidemiological studies. It can be ruled out with reasonable confidence that this relationship is explained by chance, bias or confounding.

Moderate evidence of a causal association (++):
A causal relationship is likely. A positive relationship between exposure to the risk factor and the outcome has been observed in several epidemiological studies. It cannot be ruled out with reasonable confidence that this relationship can be explained by chance, bias or confounding, although this is not a very likely explanation.

Limited evidence of a causal association (+):
A causal relationship is possible. A positive relationship between exposure to the risk factor and the outcome has been observed in several epidemiological studies. It is not unlikely that this relationship can be explained by chance, bias or confounding.

Insufficient evidence of a causal association (0):
The available studies are of insufficient quality, consistency, or statistical power to permit a conclusion regarding the presence or absence of a causal association.

Evidence suggesting lack of a causal association (-):
Several studies of sufficient quality, consistency and statistical power indicate that the specific risk factor is not causally related to the specific outcome.

**Comments:**
The classification does not include a category for which a causal relation is considered as established beyond any doubt.

The key criterion is the epidemiological evidence.
The likelihood that chance, bias and confounding may explain observed associations are criteria that encompass criteria such as consistency, number of ‘high quality’ studies, types of design etc. Biological plausibility and contributory information may add to the evidence of a causal association.
